# Quantum Dots‐caused Retinal Degeneration in Zebrafish Regulated by Ferroptosis and Mitophagy in Retinal Pigment Epithelial Cells through Inhibiting Spliceosome

**DOI:** 10.1002/advs.202406343

**Published:** 2024-10-17

**Authors:** Naying Zheng, Tingting Liao, Chuchu Zhang, Zheyang Zhang, Sen Yan, Xiaohan Xi, Fengkai Ruan, Chunyan Yang, Qingliang Zhao, Wenbo Deng, Jialiang Huang, Zi‐Tao Huang, Zhi‐Feng Chen, Xiang Wang, Qingming Qu, Zhenghong Zuo, Chengyong He

**Affiliations:** ^1^ Department of Ophthalmology in Xiang'an Hospital of Xiamen University State Key Laboratory of Cellular Stress Biology School of Life Sciences Faculty of Medicine and Life Sciences Xiamen University Xiamen Fujian 361102 China; ^2^ Department of Chemistry State Key Laboratory of Physical Chemistry of Solid Surfaces Collaborative Innovation Center of Chemistry for Energy Materials (i‐ChEM) Innovation Laboratory for Sciences and Technologies of Energy Materials of Fujian Province (IKKEM) College of Chemistry and Chemical Engineering Xiamen University Xiamen 361005 China; ^3^ State Key Laboratory of Vaccines for Infectious Diseases Center for Molecular Imaging and Translational Medicine Xiang An Biomedicine Laboratory School of Public Health Xiamen University Xiamen Fujian 361005 China; ^4^ Key Laboratory of Reproductive Health Research Fujian Province University School of Medicine Xiamen University Xiamen Fujian 361005 China; ^5^ Guangdong Key Laboratory of Environmental Catalysis and Health Risk Control Guangdong‐Hong Kong‐Macao Joint Laboratory for Contaminants Exposure and Health School of Environmental Science and Engineering Guangdong University of Technology Guangzhou 510006 China

**Keywords:** Ferroptosis, Quantum dots, Retinal degeneration, Single‐cell RNA sequencing, Spliceosome

## Abstract

Quantum dots (QDs) are widely used, but their health impact on the visual system is little known. This study aims to elucidate the effects and mechanisms of typical metallic QDs on retinas using zebrafish. Comprehensive histology, imaging, and bulk RNA sequencing reveal that InP/ZnS QDs cause retinal degeneration. Furthermore, single‐cell RNA‐seq reveals a reduction in the number of retinal pigment epithelial cells (RPE) and short‐wave cone UV photoreceptor cells (PR(UV)), accompanied by an increase in middle‐ and long‐wave cone red, green, and blue photoreceptor cells [PR(RGB)]. Mechanistically, after endocytosis by RPE, InP/ZnS QDs inhibit the expression of splicing factor *prpf8*, resulting in *gpx4b* mRNA unsplicing, which finally decrease glutathione and induce ferroptosis and mitophagy. The decrease of RPE fails to engulf the damaged outer segments of PR, possibly promoting the differentiation of PR(UV) to PR(RGB). Knockout *prpf8* or *gpx4b* with CRISPR/Cas9 system, the retinal damage is also observed. Whereas, overexpression of *prpf8* or *gpx4b*, or supplement of glutathione can rescue the retinal degenerative damage caused by InP/ZnS QDs. In conclusion, this study illustrates the potential health risks of InP/ZnS QDs on eye development and provides valuable insights into the underlying mechanisms of InP/ZnS QDs‐caused retinal degeneration.

## Graphical abstract

This study delves into the impact of QDs on the visual system, emphasizing the profound retinal degeneration caused by InP/ZnS QDs in zebrafish. The results illustrate InP/ZnS QDs‐caused retinal degeneration regulated by ferroptosis and mitophagy in RPE cells via inhibiting PRPF8‐related spliceosome. Furthermore, potential therapeutic interventions are developed, including *prpf8* or *gpx4b* overexpression, and glutathione supplement.

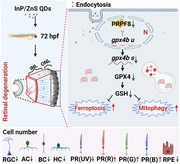

## 1 Introduction

Retinal degeneration, a leading cause of vision loss affecting millions worldwide, is characterized by progressive damage and cell death in the retina.^[^ ^1^ ^]^ Retinal degenerative damage is frequently caused by mutant proteins, hypoxia, vessel occlusion, and light sensitivity. Emerging evidence has shown that retinal degenerative damage can be caused by environmental pollutants, such as air particle matter (PM) and engineering nanoparticles. For example, PM2.5 concentrations were negatively correlated with retinal thickness from 51 710 UK Biobank participants.^[^ ^2^ ^]^ Intravitreal injection of titanium dioxide nanoparticles has been observed to thin the inner nuclear layer in the mouse retina.^[^ ^3^ ^]^

Quantum dots (QDs) are semiconductor nanomaterials generally with diameters ranging from 2 to 20 nm.^[^ ^4^ ^]^ These unique nanocrystals possess exceptional photoelectric and luminescent properties, making them highly promising for applications of light‐emitting diodes (LEDs), lasers, photodetectors, photovoltaics, displaying, bioimaging, and biomedicine.^[^ ^5^, ^6^ ^]^ The global revenue of QDs was ≈4556.2 million dollars in 2021, with a projected growth to reach 25 570 million dollars by 2028, according to a report by Global Info Research. Due to their small size and excellent optical performance,^[^ ^7^ ^]^ QDs have great potential to permeate ocular barriers and therefore have been used in many ophthalmological studies, including imaging, and tracking ocular cells,^[^ ^8^ ^]^ antibacterial agents,^[^ ^9^ ^]^ and ocular disease therapy.^[^ ^10^ ^]^ For instance, CdSe/ZnS QDs are applied to long‐term track endothelial progenitor cells in a choroidal neovascularization rat model.^[^ ^8^ ^]^ But there are numerous studies have shown that QDs can induce cardiac,^[^ ^11^ ^]^ hepatic,^[^ ^12^ ^]^ renal,^[^ ^13^ ^]^ and reproductive toxicities.^[^ ^14^ ^]^ For example, single injection with 50 mg/kg CdSe QDs via tail vein caused kidney injury in mice possibly through inhibiting antioxidant pathway.^[^ ^15^ ^]^ Our recent study showed that 1 mg L^−1^ CdSe/ZnS QDs exposure to zebrafish embryos for 72 hours post‐fertilization (hpf) could induce pericardial edema.^[^ ^11^ ^]^ Along with the extensive applications, QDs would be omnipresent in manufacturing shops, consumer products, medical devices, and especially ophthalmological medicines. However, the retinal effects of QDs are often overlooked,^[^ ^16^ ^]^ and there is little research on biosafety of QDs on the visual system. For example, An et al. found that graphene oxide (GO) QDs incrassated corneal stromal layer, and induced cell apoptosis in the cornea in mice.^[^ ^17^ ^]^ The ocular health impacts of QDs have raised increasing concerns by scholars and the public.

In this study, we aimed to investigate the impacts of several typical QDs on eye development and its mechanisms in a zebrafish (*Danio rerio*) model. Zebrafish is an excellent ocular vertebrate model.^[^ ^18^, ^19^ ^]^ By 72 hpf, zebrafish retinas are fully developed, and exhibit similar morphology and function to the human retina,^[^ ^20^ ^]^ and the complete structure (including cornea, etc.) and optic response of the eye is fully developed at 168 hpf.^[^ ^21^ ^]^ Our findings revealed that the retinal degenerative damage was caused by many types of QDs, especially by InP/ZnS QDs, one kind of widely used metallic QDs. Consequently, we focused on InP/ZnS QDs as a representative one. Thereafter, we employed single‐cell RNA‐seq (scRNA‐seq) and subsequent experiments, such as CRISPR/Cas9 knockout and mRNA overexpression, to elucidate the effects and mechanisms of this InP/ZnS QDs‐induced retinal degenerative damage. Our results not only provided a comprehensive overview of the changes occurring in all types of zebrafish retina cells after exposure to InP/ZnS QDs, but also illustrated the underlying mechanisms of InP/ZnS QDs‐caused retinal degeneration.

## 2 Results and Discussion

### 2.1 Characterizations of QDs

**Figure 1:**
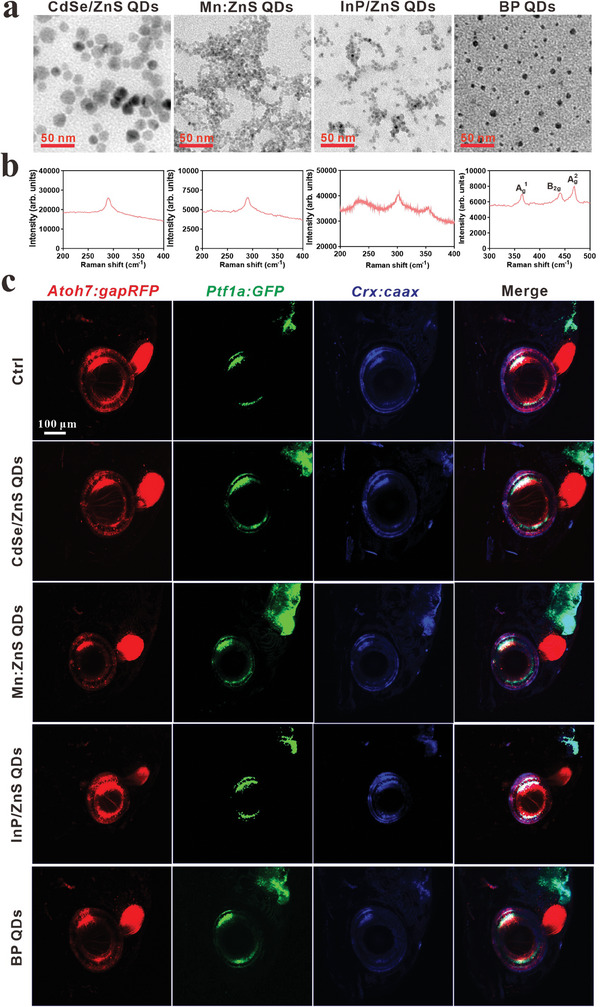
Characterizations of QDs and screening their impacts on the eye development of zebrafish. a) Representative images of QDs by TEM, scale bar, 50 nm. b) The Raman intensity of QDs. c) The lateral views of the effect of QDs (1 mg L^−1^) on retinal development in Tg (*Atoh7:gapRFP::Ptf1a:GFP::Crx:CFPcaax*) zebrafish at 168 hpf. The retinal ganglion cell layer (GCL) is labeled by red fluorescence protein (RPF), the inner nuclear layer (INL, including AC and HC) is labeled by green fluorescent protein (GFP), and cyan fluorescent protein (CFP), BC is labeled by CFP, and outer nuclear layer (ONL, composed of PR) labeled by RFP and CFP. Scale bar, 100 µm.

In the present study, we selected four kinds of QDs [CdSe/ZnS PEG‐COOH QDs, InP/ZnS PEG‐COOH QDs, Mn‐doped ZnS (Mn:ZnS) QDs, and black phosphorus (BP) QDs] considering their current use and next‐generation applications in LEDs, photovoltaics, and displaying, and so on.^[^ ^22^, ^23^, ^24^, ^25^ ^]^ The morphology and sizes of these QDs were examined by transmission electron microscopy (TEM). As shown in **Figure** **1**a, all of them were spherical and the diameters were 16.73 ± 3.1, 2.21 ± 2.5, 6.21 ± 2.1, and 5.61 ± 2.4 nm, respectively (Table S1, Supporting Information). The hydrophilic dynamic sizes in culture medium were 90.61 ± 32.39, 618.27 ± 17.62, 120.20 ± 45.51, 247 ± 23.58 nm, respectively, and all the zeta potentials were negative (−17.93 ± 0.85, −14.57 ± 1.19, ‐7.0 ± 1.38, −0.30 ± 0.84 mV, respectively). Raman shift was also conducted to validate the components of QDs, the characteristic peaks were similar to previous studies (Figure 1b).^[^ ^26^, ^27^, ^28^, ^29^ ^]^

### 2.2 QD Embryonic Exposure Decreased the Eye Size of Zebrafish

We chose 0, 0.1, and 1 mg L^−1^ of these QDs exposed to zebrafish from 0.5 to 72 hpf, and compared their effect to zebrafish retina. The results showed that the survival rate was not affected, but the eye size was significantly decreased and it was mostly altered in InP/ZnS QDs treatment group (Figure S1a,b, Supporting Information). To explore the potential bioeffects of these selected QDs on the retinal lamination and neuronal differentiation, we employed the Spectrum of Fates (SoFa) transgenic (Tg) (*Atoh7:gapRFP::Ptf1a:GFP::Crx:CFPcaax*) zebrafish.^[^ ^30^ ^]^ This model incorporates three fluorescent protein genes fused with promoters specific to one or more types of retinal cells, enabling simultaneous visualization of these cells. As a crucial pro‐neuronal transcription factor, *atoh7* serves as a marker for the retinal ganglion cells (RGCs) in the zebrafish retina.^[^ ^31^, ^32^ ^]^ *Ptf1a* specifically expresses in the amacrine cells (AC) and horizontal cells (HC) of the retina.^[^ ^33^ ^]^ *Crx* is a marker for bipolar cells (BC) and photoreceptor (PR) cells.^[^ ^34^ ^]^ Therefore, RGC are labeled by red fluorescence protein (RFP). The inner nuclear layer (INL) is composed of AC, HC, and BC. In INL, AC, and HC are labeled by green fluorescent protein (GFP) and cyan fluorescent protein (CFP). BC are labeled by CFP. The outer nuclear layer is the nuclei of PR, which is labeled by RFP and CFP.

The SoFa embryos were exposed to these four types of QDs at 1 mg L^−1^ from 0.5 to 72 hpf, and we observed retinal toxicity at 168 hpf. As shown in Figure 1c, the eye size was significantly decreased in the CdSe/ZnS, InP/ZnS, and Mn:ZnS QDs groups. More specially, these QDs shortened the RGC layer, and the thickness of ONL and INL was significantly decreased by InP/ZnS QDs (Figure S1c,d, Supporting Information). In short, InP/ZnS QDs most severely affected the retinal development of zebrafish (Figure 1c). The shortening of eye diameters and thinning of retinal layers caused by these metallic QDs are typical symptoms of retinal degeneration.^[^ ^35^ ^]^ Therefore, we deduced that exposure to metallic QDs might induce retinal degeneration in zebrafish.

### 2.3 InP/ZnS QDs Exposure Caused Zebrafish Retinal Degenerative Damage

**Figure 2:**
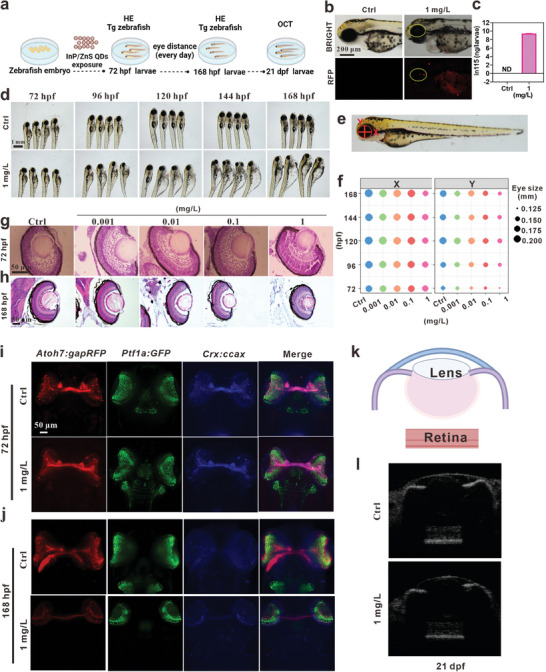
InP/ZnS QDs embryonic exposure caused retinal degeneration in zebrafish. a) Schematic illustration of the experimental design. b) Representative images of the distribution of InP/ZnS QDs on zebrafish eye using fluorescence microscopy. Scale bar, 200 µm. c) Determination of indium element content in the retina of zebrafish by ICP‐MS, n = 6, ND: No Detected. d) Representative images of zebrafish from 72 to 168 hpf after exposure. Scale bar, 1 mm. e) Schematic diagram of horizontal (X) and vertical (Y) axes of zebrafish eye. f) The lengths of X and Y axes in the zebrafish eye at 72–168 hpf, n = 20. Representative images of zebrafish retinal histology at 72 hpf g) and 168 hpf h) as assessed by HE staining. Scale bar, 50 µm. i‐j) Representative images of the retinas of Sofa zebrafish at 72 hpf i) and 168 hpf j) using a light‐sheet microscope. The retinal ganglion cell layer (GCL) is labeled by RPF, the inner nuclear layer (INL, composed of AC and HC) is labeled by GFP and CFP, BC is labeled by CFP, and the outer nuclear layer (ONL, composed of PR) labeled by RFP and CFP. k) Scheme of zebrafish eye. l) The representative images of zebrafish eye by optical coherence tomography (OCT) at 21 dpf. The data are presented as mean ± SE. One‐way ANOVA followed by *Duncan test* was used to assess the statistical significance. Significant change among different groups (*p* < 0.05) is indicated by the different letters on the bar.

In order to understand the underlying mechanisms of the retinal developmental damage caused by metallic QDs, we selected InP/ZnS QDs as a representative for the following experiments. In zebrafish, RGCs, the first type of retinal cells, appear at 36 hpf,^[^ ^36^ ^]^ and other types of retinal cells are fully differentiated till 72 hpf.^[^ ^37^ ^]^ A review summarized that the exposure concentrations of QDs to zebrafish range 0.005 from 100 mg L^−1^.^[^ ^38^ ^]^ Considering the great toxicity of 10 and 100 mg L^−1^ QDs in those studies, we set the concentrations at 0.001, 0.01, 0.1, and 1 mg L^−1^, and exposed from 0.5 to 72 hpf (**Figure** **2**a). The exposure of InP/ZnS QDs decreased the embryo movement at 24 hpf, delayed the embryo hatching from 48 to 60 hpf, and increased the heart rate and malformation rate at 72 hpf in a concentration‐dependent manner (Figure S2a,b,c,f, Supporting Information). The expression levels of zebrafish hatching enzyme genes (*zhe1* and *zhe2*) were significantly decreased after InP/ZnS QDs treatment (Figure S2d,e, Supporting Information). The hatching delay led us to consider whether InP/ZnS QDs caused developmental retardation in zebrafish. Thus, we measured the body length of the zebrafish and found no significant change (Figure S2g, Supporting Information). Consistently, the developmental stages of zebrafish were not affected by InP/ZnS QDs exposure (Figure S2h,i, Supporting Information), which further demonstrated that InP/ZnS QDs did not delay the development of zebrafish.

Meanwhile, we found the distribution of InP/ZnS QDs mainly on the yolk and eye at 72 hpf through their red fluorescent signals using a fluorescence microscope (Figure 2b). In addition, we isolated the zebrafish retinas at 72 hpf for inductively coupled plasma‐mass spectrometry (ICP‐MS), and found that InP/ZnS QDs exposure increased the indium element content (Figure 2c). The size of the zebrafish eye is usually evaluated using the diameters of the horizontal (X) and vertical (Y) axes (Figure 2e). We examined the eye size from 72 to 168 hpf and found that both X and Y axes were significantly shorter in InP/ZnS QDs‐treated group than those in control (Figure 2d,f). Moreover, the retinas also went through degenerative damage after InP/ZnS QDs exposure as detected by hematoxylin‐eosin (HE) staining at 72 and 168 hpf (Figure 2g,h). InP/ZnS QDs exposure decreased the thicknesses of the whole retina, RPE, ONL, INL, ganglion cell layer (GCL), and the diameter of lens (Figure S3a–m, Supporting Information) in a concentration‐dependent manner.

SoFa Tg zebrafish embryos were also exposed to 1 mg L^−1^ InP/ZnS QDs from 0.5 to 72 hpf. We imaged the retinas at the dorsal view and found that the cell number of INL was dramatically decreased at 72 hpf (Figure 2i) and the number of all retinal cells was reduced at 168 hpf (Figure 2j). Optical coherence tomography (OCT) imaging is a rapid and non‐invasive examination for the retina, which has been widely used by opticians.^[^ ^39^ ^]^ Herein, we evaluated the retinal morphology at 21 days post‐fertilization (dpf) using OCT, which showed that the thickness of the retina was significantly thinner in InP/ZnS QDs‐exposed larvae (Figure 2k,l). In short, these data suggested that InP/ZnS QDs embryonic exposure disturbed eye development and caused retinal degeneration in zebrafish.

### 2.4 Bulk RNA‐seq Revealed the Potential Mechanisms of Retinal Degeneration Caused by InP/ZnS QDs in Zebrafish

**Figure 3:**
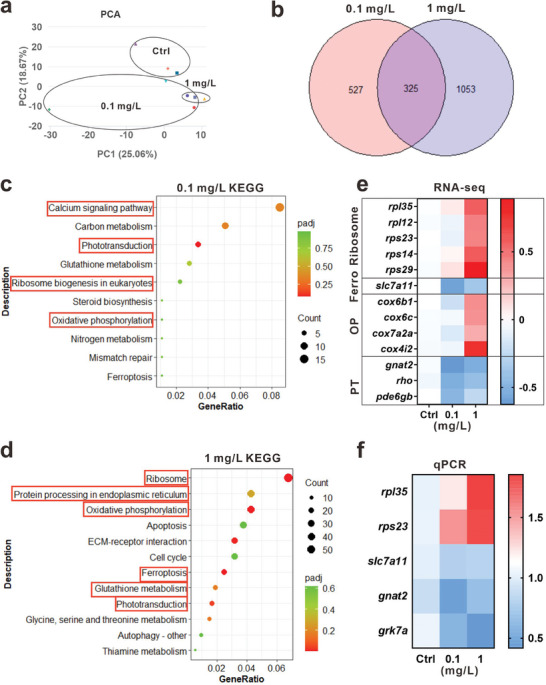
InP/ZnS QDs embryonic exposure induced many types of cell injury and impaired phototransduction in zebrafish retinas using bulk RNA‐seq. After exposure to InP/ZnS QDs, the zebrafish eyes at 72 hpf were collected to perform RNA‐seq, n = 3. a) The PCA result of control, 0.1 and 1 mg L^−1^ InP/ZnS QDs. b) Venn diagram of the overlapped DEGs in the 0.1 and 1 mg L^−1^ groups compared to the control with 0.5‐fold cutoff and *p* < 0.05. c,d) KEGG pathway analysis of the DEGs in 0.1 c) and 1 mg L^−1^ groups d), respectively. e) The heatmap of DEGs in the ribosome, ferroptosis, oxidative phosphorylation, and phototransduction pathways. Ferro: ferroptosis, OP: oxidative phosphorylation, PT: phototransduction. f) The qPCR was performed to validate the DEGs determined from RNA‐seq, n ≥ 3.

To explore the underlying biological mechanisms of InP/ZnS QDs‐induced retinal degenerative damage, zebrafish embryos were exposed to 0.1 and 1 mg L^−1^ InP/ZnS QDs from 0.5 to 72 hpf. Then, we separated the eyes to perform bulk RNA‐seq. The principal component analysis (PCA) showed that 0.1 and 1 mg L^−1^ InP/ZnS QDs‐exposed groups significantly different from control (**Figure** **3**a). Moreover, with the |log2FoldChange| > 0.5 and *p* < 0.05 cutoff, there were 852 and 1378 differentially expressed genes (DEGs) in 0.1 and 1 mg L^−1^ InP/ZnS QDs‐exposed groups, respectively. There were 325 overlapped DEGs as shown in the Venn diagram (Figure S4a,b, Supporting Information; Figure 3a,b). Next, we performed the Kyoto Encyclopedia of Genes and Genomes (KEGG) analysis. In 0.1 mg L^−1^ group, some pathways were enriched, such as the calcium signaling pathway, phototransduction, oxidative phosphorylation, ribosome biogenesis in eukaryotes (Figure 3c). Besides, there were some other pathways enriched in 1 mg L^−1^ group, including ribosome, protein processing in the endoplasmic reticulum (ER), ferroptosis, glutathione metabolism, etc. (Figure 3d). Through the heatmap of DEGs from RNA‐seq, we found that InP/ZnS QDs exposure induced many types of injury, including ribosome, ferroptosis, oxidative phosphorylation, and impaired phototransduction in zebrafish retinas (Figure 3e). Furthermore, some of the DEGs were validated by qPCR (Figure 3f). In line with our results, a previous study reported that CdTe QDs caused ferroptosis through oxidative stress and inflammation in macrophages.^[^ ^40^ ^]^ These results indicated that InP/ZnS QDs triggered many types of cell injury and weakened the phototransduction in the retinas of zebrafish. However, it was still unknown what had happened in each type of retinal cells.

### 2.5 ScRNA‐Seq Analysis Revealed the Changes and Fates of All Types of Retinal Cells in Zebrafish after InP/ZnS QDs Exposure

**Figure 4:**
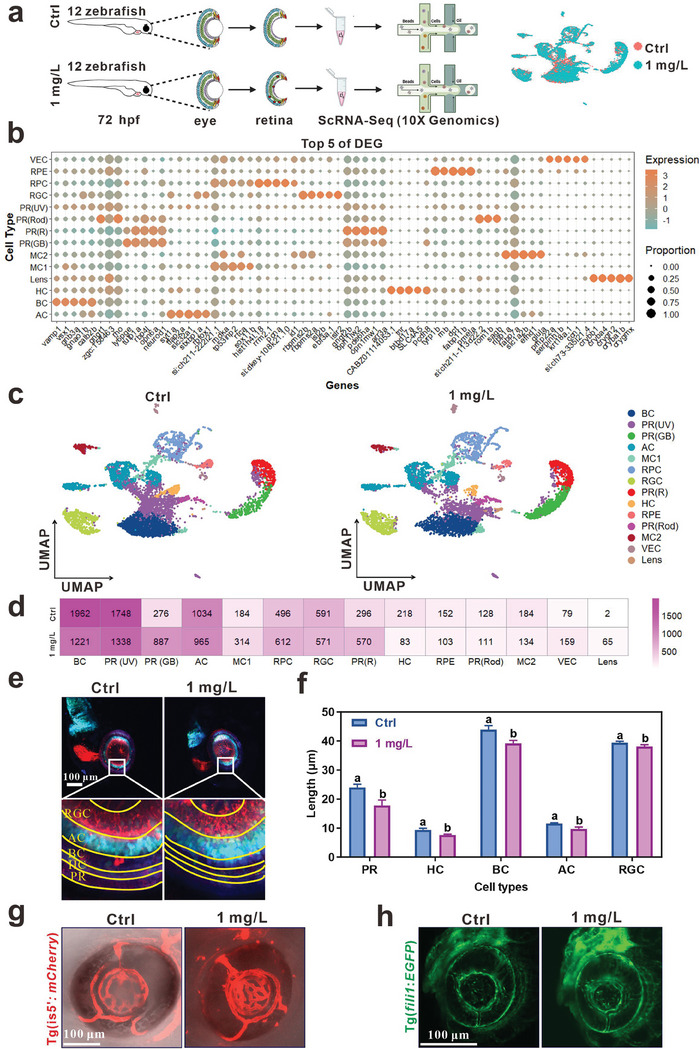
ScRNA‐seq analysis displayed the retinal degenerative damage of zebrafish exposed to InP/ZnS QDs at single cell level. a) Schematic illustration of the scRNA‐seq experimental design. We dissociated the retinas from zebrafish exposed to InP/ZnS QDs from 0.5 to 72 hpf (24 retinas of 12 larvae from each group). b) The top five DEGs of each cluster. c) UMAP plots show the clustering of qualified zebrafish eyes at 72 hpf. Distinct cell types are marked by different colors. d) The cell number of different clusters between control and InP/ZnS QDs‐exposed group. e) The representative images of the retina of Tg (*Atoh7:gapRFP::Ptf1a:GFP::Crx:CFPcaax*) zebrafish. f) The thicknesses of different layers of zebrafish retina were measured, n = 4. g‐h) The representative images of blood vessels in Tg (*is5’:mCherry*) g) and Tg (*fli1:EGFP*) h) zebrafish. Scale bar, 100 µm. The data are presented as mean ± SE. Significant change among different groups (*p* < 0.05) is indicated by the different letters on the bar.

ScRNA‐seq analysis was employed to uncover the changes and fates of all retinal cell types in zebrafish following exposure to 1 mg L^−1^ InP/ZnS QDs. We dissociated the retinas from zebrafish exposed to InP/ZnS QDs from 0.5 to 72 hpf (24 retinas of 12 larvae from each group), and then performed scRNA‐seq (10x Genomics) (**Figure** **4**a, left panel). Libraries of control and InP/ZnS QDs group were individually sequenced to a mean depth of 391 961 731 and 396 968 933 reads per library, with a median of ≈1758 and 2242 unique molecular identifiers (UMI) and ≈928 and 1047 genes per cell, respectively. We conducted raw data processing using CellRanger and then removed low‐qualified cells using the Seurat R package (http://satijalab.org/seurat/). The datasets obtained of zebrafish retinas contained 7350 and 7133 high‐quality single‐cell transcriptomes in control and InP/ZnS QDs‐exposed group, respectively. To characterize the atlas of the whole retinal cells, the datasets were integrated using canonical correlation analysis (CCA) with unsupervised clustering and visualized by uniform manifold approximation and projection (UMAP) (Figure 4a, right panel). Based on the top five DEGs of different retinal cells,^[^ ^37^ ^]^ ten types of cells that fell into 14 distinct cell clusters were identified: retinal progenitor cells (RPC), RGC, BC, AC, HC, and PR (including four subpopulations), as well as some other none‐retinal neuron cells, such as Müller cells (MC, including two subpopulations: MC1 and MC2), retinal pigment epithelial cells (RPE), vascular endothelial cells (VEC), and lens epithelial cells (Lens) (Figure 4b; Figure S5, Supporting Information). The PR cells are specialized light‐detecting neurons, consisting of an outer segment, an inner segment, the nuclear part, and synapses.^[^ ^41^ ^]^ The outer segment has a sensory cilium with G protein‐coupled receptors (Opsins, OPNs) that are sensitive to photons.^[^ ^42^ ^]^ PR cells can be divided as rod and cone cells. In contrast to mammalian retinas, the cone cells are much more than the rod cells in zebrafish with ≈92% in larvae and ≈60% in adults.^[^ ^43^ ^]^ In zebrafish, there are one type of rod cells [PR (Rod)] and four distinct subtypes of cone cells: the short‐wave sensitive cones [PR(UV)], two middle‐wave sensitive cones [including PR green and blue, PR(GB)], and one long‐wave sensitive cones [PR red, PR(R)].^[^ ^18^ ^]^ In our study, the PR cells were clustered into four subpopulations including PR(Rod), PR(UV), PR(GB), and PR(R).

Notably, the number of main retinal cells, including BC, PR(UV), HC, and RPE, was decreased from 1962, 1748, 218, and 152 to 1221, 1338, 83, and 103 after exposure, respectively. The number of PR(GB) and PR(R) was increased from 276 and 296 to 887 and 570, respectively (Figure 4c,d). Then, we employed SoFa Tg zebrafish embryos exposure to InP/ZnS QDs from 0.5 to 72 hpf. We imaged the retinas at the lateral view using confocal microscopy and measured the thickness of different retinal layers. Consistent with the number change, the thickness of each retinal layer was significantly reduced (Figure 4e,f).

Besides the main types of retinal neurons, the number of VEC was increased from 79 to 159 (Figure 4c,d). To confirm this finding, we employed two zebrafish lines, Tg (*is5:mCherry*) and Tg (*fli1: EGFP*),^[^ ^44^ ^]^ both of which label the VEC, to investigate the detailed vasculopathy. As shown in Figure 4g,h, we observed the vessel hyperplasia in the eye of zebrafish exposed to InP/ZnS QDs, compared to that in control (Figure S6, Supporting Information). Abnormal angiogenesis is one of the hallmarks of many vision‐loss diseases. Vessel hyperplasia happens in many retinal degenerative diseases, such as retinitis pigmentosa (RP), diabetic retinopathy (DRP), ischemic retinopathy, and maculopathy.^[^ ^45^, ^46^ ^]^ These results further demonstrate that embryonic exposure to InP/ZnS QDs induced retinal degeneration in zebrafish through different destruction of retinal cells.

### 2.6 InP/ZnS QDs Exposure Altered the Number and Function of Different Types of PR Cells in Zebrafish

**Figure 5:**
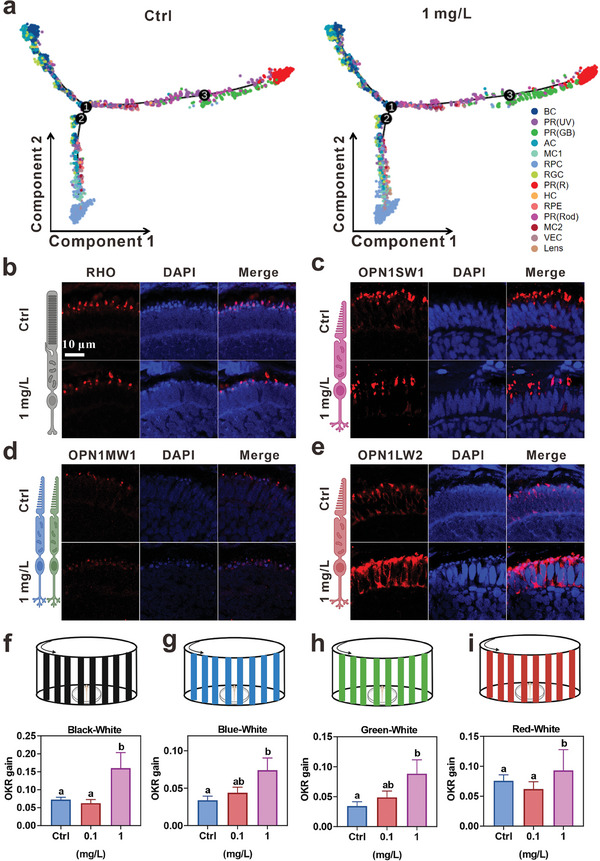
The number and function alteration of PR cells in zebrafish after exposure to InP/ZnS QDs. a) Pseudotime trajectory analysis of retinal cell development with Monocle. Each dot is a single cell and its color is agreement with its cluster assignment. b–e) The representative images of PR(Rod), PR(UV), PR(GB), and PR(R) of zebrafish at 72 hpf stained by RHO b), OPN1SW1 c), OPN1MW1 d), and OPN1LW2 e), respectively. f–i) The scheme and gain of optokinetic response (OKR) of 168‐hpf zebrafish in Black‐White f), Blue‐White g), Green‐White h), and Red‐White stripes i), n ≥ 5. The data are presented as mean ± SE. Significant change among different groups (*p* < 0.05) is indicated by the different letters on the bar.

During the development of zebrafish retina, RPC starts at 12 hpf, and then differentiates into RGC at 36 hpf, and other retinal cells including AC, BC, HC, and MC at 48 hpf, PR at 60 hpf, and all types of retinal cells are completely differentiated at 72 hpf.^[^ ^37^ ^]^ In order to investigate whether InP/ZnS QDs exposure led to retinal degeneration by affecting the differentiation of retinal cells, we used UMAP dimensionality reduction to track retinal cell developmental trajectory using Monocle with default parameters.^[^ ^47^ ^]^ As shown in **Figure** **5**a, the retinal cell differentiation was not affected by InP/ZnS QDs exposure, which was consistent with the developmental stage analysis in Figure S2 (Supporting Information).

As stated above, we defined four types of PR cells [PR(Rod), PR(UV), PR(GB), and PR(R)] from scRNA‐seq analysis. The number of PR(Rod) and PR(UV) cells was sharply decreased by InP/ZnS QDs exposure, but that of PR(GB) and PR(R) was increased. Ridgeplot analysis presented the expression level of marker genes, including *rho*, *opn1sw1*, *opn1mw1*, and *opn1lw2*, for PR(Rod), PR(UV), PR(GB), and PR(R), respectively. The mountain peaks of these marker genes confirmed the number changes of PR cells (Figure S7a–d, Supporting Information). Next, we conducted immunofluorescence to validate the changes of PR cell number. Consistently, the RHO [a marker for PR(Rod)] and OPN1SW1 [a marker for PR(UV)] positive cells were dramatically reduced in InP/ZnS QDs exposure group than those in control (Figure 5b,c; Figure S7e,f, Supporting Information); and the OPN1MW1 [a marker for PR(GB)] and OPN1LW2 [a marker for PR(R)] positive cells were increased (Figure 5d,e; Figure S7g,h, Supporting Information).

It is well known that PR cells are responsible for the conversion of light signals to electrical signals in the process of phototransduction.^[^ ^48^ ^]^ Due to the larvae lack of swimming capacity at 72 hpf, we stimulated larvae at 168 hpf with purple, blue, green, and red light, respectively, to investigate the physiological functions of different PR cells. In agreement with the number changes of different types of cone cells, the blue, green, and red lights, but not purple, significantly accelerated the swimming movements of zebrafish in a concentration‐dependent manner (Figure S7i–m, Supporting Information). These results suggested that InP/ZnS QDs exposure elevated the visual sensitivity to middle‐ and long‐wave lights in PR cone cells. Furthermore, we selected zebrafish in the control, 0.1 and 1 mg L^−1^ groups for optokinetic response (OKR) test, and found that 1 mg L^−1^ InP/ZnS QDs caused larvae to be more sensitive to Black‐White stripes (Figure 5f), Blue‐White stripes (Figure 5g), Green‐White stripes (Figure 5h), and Red‐White stripes (Figure 5i). On the contrary, exposure to tire wear particles decreased the proliferation of PR cells, which led to disruption of phototransduction and optokinetic response (OKR) in zebrafish.^[^ ^49^ ^]^ Taken together, these findings further demonstrated that embryonic exposure to InP/ZnS QDs altered the ratio of PR cells and visual response to different wavelength lights in zebrafish.

### 2.7 The Biological Mechanisms of InP/ZnS QDs Exposure on PR Cells in Zebrafish

**Figure 6:**
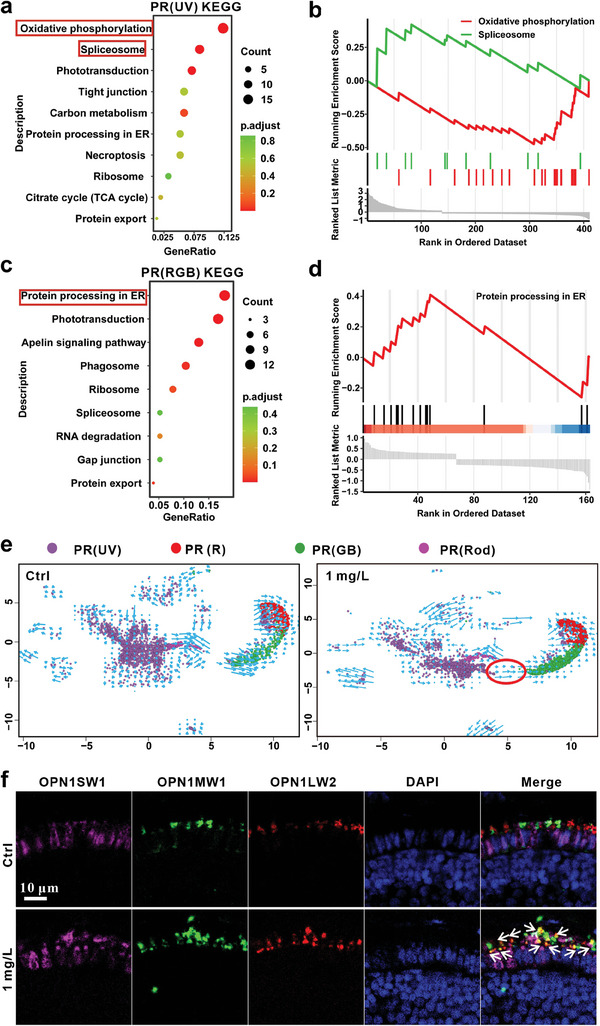
InP/ZnS QDs embryonic exposure promoted the differentiation of PR(UV) to PR(RGB) in zebrafish. a‐e) The bioinformatics analysis was based on the data from scRNA‐seq. a) KEGG pathway analysis of the DEGs in PR(UV) cells. b) The GSEA of PR(UV) cells. c) KEGG pathway analysis of the DEGs in PR(RGB) cells. d) The GSEA of PR(RGB) cells. e) The single‐cell RNA velocity of four types of PR cells in the control and InP/ZnS QDs group, respectively, using by cell velocity. The red cycle indicates the differentiation of PR(UV) to PR(RGB). f) Fluorescence triple staining assay (TSA) was used to track PR(UV), PR(R), and PR(GB), respectively. The white arrowheads display the co‐location of PR(UV) with PR(R) or PR(GB), suggesting the differentiation of PR(UV) to PR(RGB) caused by InP/ZnS QDs exposure. The images were captured by confocal microscopy. Scale bar, 10 µm.

We analyzed the enriched pathways of DEGs using KEGG and gene set enrichment analysis (GESA) to investigate the molecular mechanisms. As shown in **Figure** **6**a,b, InP/ZnS QDs exposure upregulated the spliceosome pathway, but downregulated the oxidative phosphorylation pathway in PR(UV) cells. The upregulation of spliceosome pathway would cause the disorder of RNA splicing and maturation.^[^ ^50^ ^]^ RNA velocity has been developed to characterize the dynamics of spliced and unspliced mRNAs to describe the change rate of gene expression for each gene at a specific time point.^[^ ^51^ ^]^ Thereafter, we performed the RNA velocity analysis of PR cells and found that a great number of short‐wave cone cells [PR(UV)] were differentiated into the middle‐ and long‐wave ones [PR(GB) and PR(R)] (Figure 6e).

The middle‐ and long‐wave cone cells [PR(GB) and PR(R)] displayed similar changes in cell number and RNA velocity, thus they were combined as PR(RGB) for further analysis. KEGG and GESA results showed that the pathway of protein procession in endoplasmic reticulum (ER) was significantly upregulated, indicating the increase in protein synthesis and processing, which was matched with the increase of PR(RGB) number (Figure 6c,d). Further, fluorescence triple staining assay (TSA) was used to label PR(UV), PR(R), and PR(GB), respectively. The result presented that parts of PR(UV) cells co‐located with PR(R) or PR(GB) in the InP/ZnS QDs‐exposed group, but not in control, which demonstrated that InP/ZnS QDs exposure promoted the differentiation of PR(UV) to PR(RGB) in zebrafish (Figure 6f). The disorder of differentiation and dysfunction of PRs was considered to contribute to the retinal degeneration in zebrafish caused by InP/ZnS QDs.

### 2.8 InP/ZnS QDs‐induced Zebrafish Retinal Degeneration Possibly Mediated by Disruption of the Spliceosome, Leading to Activation of Ferroptosis and Mitophagy in RPE

**Figure 7:**
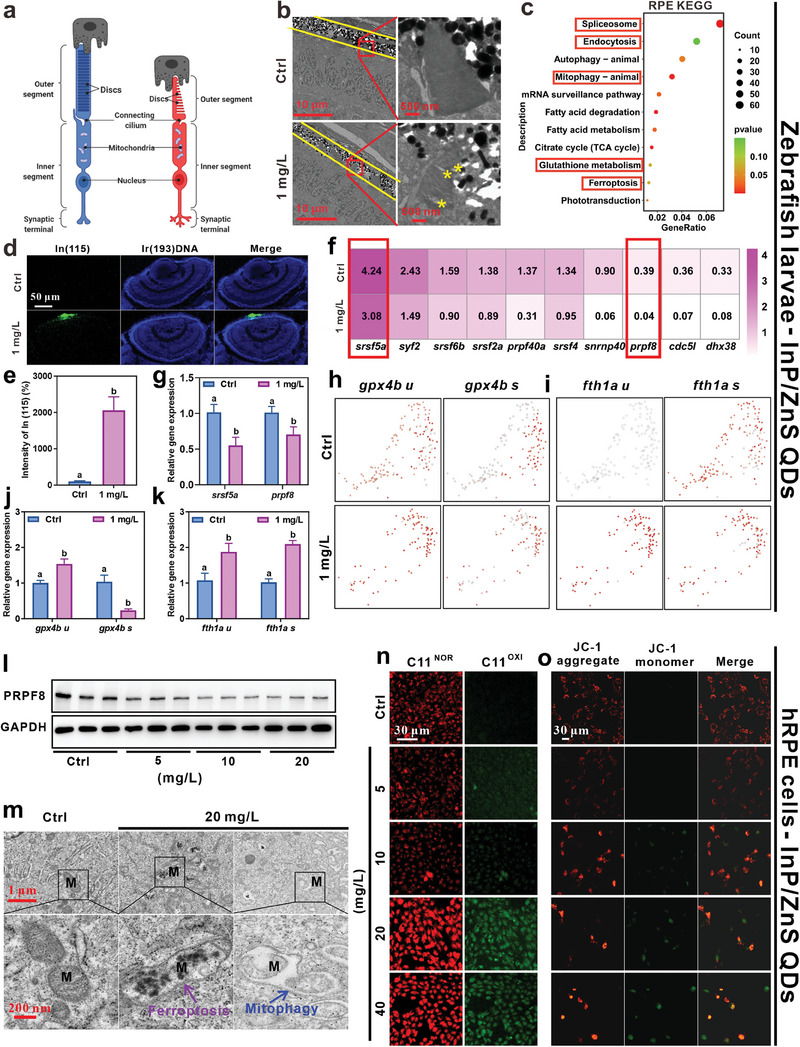
InP/ZnS QDs, endocytosed by RPE cells, caused retinal degenerative damage involved in mitophagy and ferroptosis due to spliceosome inhibition. a) The scheme shows the interaction between RPE and PR.^[^ ^52^, ^53^ ^]^ b) The representative TEM images of the structure of RPE and the outer segment (OS) of PR of zebrafish at 72 hpf. The yellow asterisk indicates the damaged OS discs. The scale bar is 10 µm and 500 nm, respectively. c) KEGG pathway of the DEGs of RPE between control and InP/ZnS QDs exposure group based on the scRNA‐seq data. d) The representative images of mass spectrometry imaging (MSI) showed InP/ZnS QDs endocytosed by RPE. The In(115) was marked with green, and DNA (cell nuclei) stained with Ir(193) was marked with blue. The scale bar is 50 µm. e) The signal intensity of In(115) was determined using by ImageJ, n = 6. f, g) The expression level of splicing factors in RPE was analyzed by Rstudio f) and qPCR g), n = 4. h,i) The spliced, and unspliced mRNA level of *gpx4b* h) and *fth1a* i) were examined by Rstudio and qPCR j,k). l) The relative protein expression level of PRPF8 in hRPE cells after different doses of InP/ZnS QDs treatment. m) The representative images of the normal mitochondria left), ferroptosis center) and mitophagy right) mitochondria in control and InP/ZnS QDs exposure group. The purple arrow indicates ferroptosis, the blue arrow indicates mitophagy, scale bar is 1 µm and 200 nm, respectively. n) the C11 BODIPY staining measured the lipid peroxidation level in control and InP/ZnS QDs exposure group, scale bar is 30 µm. o) The representative images of the JC‐1 aggregate and JC‐1 monomer in control and InP/ZnS QDs exposure group. The scale bar is 30 µm. The data are presented as mean ± SE. Significant change among different groups (*p* < 0.05) is indicated by the different letters on the bar.

The RPE layer in the retina forms the outermost aspect lying between the retinal neurons and the choroid.^[^ ^52^ ^]^ The apical microvilli of RPE cells envelop the PR‐damaged outer segment (OS) and separate the distal discs from the remaining stack. These shed discs are phagocytosed and degraded within the RPE (depicted in **Figure** **7**a).^[^ ^53^ ^]^ Using TEM, we observed that RPE wrapped the damaged OS discs in the control, but not in InP/ZnS QDs group, indicating that the damaged OS discs were not timely phagocytosed after exposure (Figure 7b). Consistently, the number of RPE was decreased from 152 to 103 after InP/ZnS QDs exposure using scRNA‐seq (Figure 4d). These results provided a rational explanation that the reduce of RPE caused the increase of OS length, which might regulate the differentiation of PR(UV) to PR(RGB) in zebrafish exposure to InP/ZnS QDs.

We then performed the KEGG analysis for RPE. As shown in Figure 7c, many pathways were enriched, such as endocytosis, spliceosome, glutathione metabolism, and many types of cell death including mitophagy and ferroptosis. We then conducted mass spectrometry imaging (MSI), which confirmed that InP/ZnS QDs were mainly endocytosed by RPE (Figure 7d,e). Epithelial cells have been reported to be able to engulf exogenous substances, which is similar with immune cells.^[^ ^54^ ^]^ Therefore, we deduced that InP/ZnS QDs endocytosed by RPE cells might be the main exposure pathway of InP/ZnS QDs in zebrafish retinas.

It is still unclear how InP/ZnS QDs reduced the cell number of RPE. We first focused on the spliceosome pathway in RPE (Figure 7c). Consistently, the gene expression levels of some splicing factors, such as *prpf8* and *srsf5a*, were decreased by InP/ZnS QDs exposure determined by both Rstudio and qPCR (Figure 7f,g), which suggested that InP/ZnS QDs disrupted the spliceosome in RPE. Ferroptosis, glutathione metabolism, and mitophagy pathways were also enriched (Figure 7c). Through RNA velocity and qPCR, we found that the unspliced levels of anti‐ferroptosis gene, *gpx4b*, and ferroptosis marker gene, *fth1a*, were increased in the InP/ZnS QDs treated group (Figure 7h–k). The spliced levels of *gpx4b* were decreased (Figure 7h,j), but the spliced *fth1a* was increased (Figure 7i,k), indicating that InP/ZnS QDs exposure could cause ferroptosis in RPE cells which were regulated by the spliceosome. However, the unspliced levels of mitophagy‐related genes were not altered (Figure S8a,b, Supporting Information).

The RPE cells in zebrafish contain a high concentration of melanin particles, rendering structures such as mitochondria unobservable.^[^ ^55^ ^]^ Therefore, human retinal pigment epithelial cells (hRPE) were exposed to InP/ZnS QDs for structural observation. TEM results revealed that InP/ZnS QDs were endocytosed by hRPE cells, resulting in condensed mitochondrial membrane densities and lysosomal phagocytosis of mitochondria, indicative of ferroptosis^[^ ^56^ ^]^ and mitophagy,^[^ ^57^ ^]^ respectively (Figure 7m; Figure S9a, Supporting Information). Consistent with the zebrafish experiment in vivo, InP/ZnS QDs treatment decreased the PRPF8 protein expression in hRPE cells (Figure 7l, Figure S9b, Supporting Information). JC1 and BODYPI C11 staining further verified mitophagy and ferroptosis in hRPE cells, respectively (Figure 7n,o). These results suggested that InP/ZnS QDs were endocytosed by RPE cells, leading to mitophagy and ferroptosis by disrupting the splice factors.

### 2.9 InP/ZnS QDs Inhibited *prpf8*, Subsequently Downregulating *gpx4b* Splicing and Glutathione, Leading to Ferroptosis and Mitophagy in RPE

**Figure 8:**
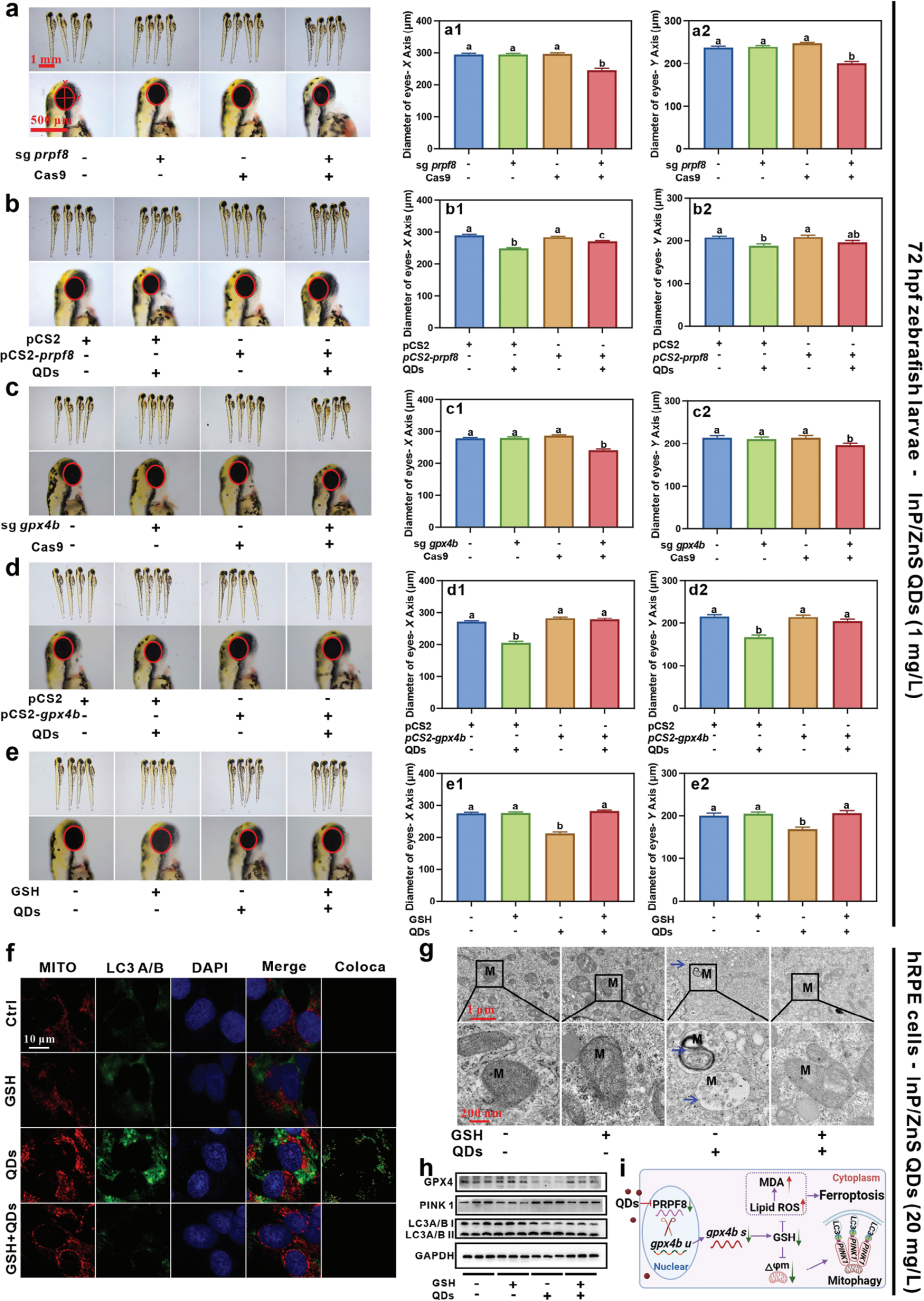
Mechanism of retinal degeneration induced by InP/ZnS QDs in zebrafish through inhibiting *prpf8*‐related splicesome and disrupting glutathione metabolism. a) The representative wild‐type larvae images of no injection control, injection with Cas9 protein, injection with sgRNA for *prpf8*, and co‐injection of Cas9 and sgRNA for *prpf8*. The scale bar is 1 mm and 500 µm, respectively. The eye distance of in X a1) and Y a2) axis, n = 24. b) *Prpf8* overexpression alleviated the small eye size caused by InP/ZnS QDs at 72 hpf. The wild‐type embryos were divided four groups: injection of pCS2 plasmids with or without InP/ZnS QDs exposure and injection of pCS2‐*prpf8* plasmids with or without InP/ZnS QDs exposure. The scale bar is 1 mm and 500 µm, respectively. The eye distance of in X b1) and Y b2) axis, n = 20. c) The representative wild‐type larvae images of no injection control, injection with Cas9 protein, injection with sgRNA for *gpx4b*, and co‐injection of Cas9 and sgRNA for *gpx4b*. The scale bar is 1 mm and 500 µm, respectively. The eye distance of in X c1) and Y c2) axis, n = 18–20. d) *gpx4b* overexpression alleviated the small eye size caused by InP/ZnS QDs at 72 hpf. The wild‐type embryos were divided four groups: injection of pCS2 plasmids with or without InP/ZnS QDs exposure and injection of pCS2‐*gpx4b* plasmids with or without InP/ZnS QDs exposure. The scale bar is 1 mm and 500 µm, respectively. The eye distance of in X d1) and Y d2) axis, n = 19–20. e) GSH rescued the small eye size caused by InP/ZnS QDs at 72 hpf. The wild‐type embryos were divided four groups: Control, GSH control, InP/ZnS QDs, and InP/ZnS QDs with GSH groups. The scale bar is 1 mm and 500 µm, respectively. The eye distance of in X e1) and Y e2) axis, n = 20. f) The representative images of Mito Tracker and LC3A/B in hRPE cells, Mito Tracker: red, LC3A/B: green, nuclear: blue, Coloca: colocalization of mitochondrial and LC3A/B, Scale bar is 10 µm. The representative TEM images of the structure of mitochondria hRPE cells in the four groups. The blue arrow indicates mitophagy and the scale bar is 1 µm and 200 nm, respectively g). h) The relative expression levels of proteins in hPRE cells after InP/ZnS QDs exposure with or without GSH treatment for 24 h. i) Schematic diagram of ferroptosis and mitophagy caused by InP/ZnS QDs in RPE cells. The data are presented as mean ± SE. Significant change among different groups (*p* < 0.05) is indicated by the different letters on the bar.

As stated above, InP/ZnS QDs induced retinal degeneration by triggering ferroptosis and mitophagy. However, either Fer‐1, an inhibitor of ferroptosis,^[^ ^58^ ^]^ or Brefeldin A (BFA), an inhibitor of mitophagy,^[^ ^59^ ^]^ could partially alleviate the retinal degeneration caused by InP/ZnS QDs (Figure S10, Supporting Information). Hence, our attention was directed toward the upstream spliceosome and glutathione metabolism pathways. As stated above, we found that InP/ZnS QDs inhibited spliceosome pathway in RPE. Of which, *prpf8* was downregulated close to 10 times (Figure 7f). As a core component of small nuclear ribonucleoprotein complexes (snRNP), the pre‐mRNA processing factor 8 (PRPF8) protein contributes to the activation of spliceosome.^[^ ^60^ ^]^ *PRPF8* mutations in humans and mice have been reported to be associated with retinal degeneration through inhibiting RNA splicing.^[^ ^61^ ^]^ Therefore, we first used CRISPR/Cas9 technique to mutant *prpf8* at the single‐cell phase of zebrafish. As shown in **Figures** **8**a and S11a (Supporting Information), the mutation of *prpf8* shortened the eye size at 72 hpf. In SoFa Tg zebrafish, *prpf8* mutation also reduced the thicknesses of the PR, HC, BC, AC, and RGC layers in retinas (Figure S11e,f, Supporting Information). Then, we isolated the zebrafish retinas to measure the relative expression levels of PR cone marker genes. The mutation of *prpf8* elevated the expression of *opn1mw1* and *opn1lw2* (Figure S11c,d, Supporting Information) and reduced the expression of *opn1sw1* (Figure S11b, Supporting Information), which was matched with the phenotype after InP/ZnS QDs exposure. These results showed that InP/ZnS QDs caused retinal degeneration of zebrafish possibly through inhibiting the expression of *prpf8*.

To further validate the role of *prpf8* in zebrafish retinal degeneration caused by InP/ZnS QDs, we amplified *prpf8* gene and cloned it into pCS2 plasmids to form the overexpressing plasmids (pCS2‐*prpf8*), which were then injected into embryos at the single‐cell phase, which showed that overexpression of *prpf8* partially rescued the retinal degeneration caused by InP/ZnS QDs (Figure 8b; Figure S11g–k, Supporting Information). These results indicated that InP/ZnS QDs caused retinal degeneration of zebrafish possibly through inhibiting the expression of *prpf8*.

The same methods were used to mutate and overexpress *gpx4b*, respectively. We found that *gpx4b* mutation also induced retinal degeneration as InP/ZnS QDs treatment (Figure 8c; Figure S12a, Supporting Information). Notably, overexpression of *gpx4b* completely rescued the retinal degeneration induced by InP/ZnS QDs (Figure 8d, Figure S12c, Supporting Information). Further, we found that glutathione (GSH) supplement also completely rescued the retinal degeneration caused by InP/ZnS QDs (Figure 8e, Figure S12d,e, Supporting Information). In addition, we found that GSH supplement rescued the increased levels of lipid peroxidation products malondialdehyde (MDA) in the retina (Figure S12f, Supporting Information) and the downregulation of *gpx4b* expression levels in the RPE cells caused by InP/ZnS QDs (Figure S12g, Supporting Information).

Previous studies suggested that glutathione metabolism plays a crucial role in ferroptosis^[^ ^62^ ^]^ and mitophagy.^[^ ^63^ ^]^ Does aberrant glutathione metabolism induce mitophagy and ferroptosis? We validated this hypothesis by co‐exposure of InP/ZnS QDs and GSH in hRPE cells. Co‐localization of mitochondrial probes (Mito‐Tracker) and autophagosome proteins (LC3 I/II)^[^ ^63^ ^]^ indicated that GSH rescued InP/ZnS QDs‐induced mitophagy (Figure 8f; Figure S13a, Supporting Information). TEM and protein levels further confirmed these findings (Figure 8g,h; Figure S13b–e, Supporting Information). Additionally, the decrease of MDA supported that GSH rescued InP/ZnS QDs‐induced ferroptosis (Figure S13h, Supporting Information).^[^ ^62^ ^]^ These results indicated that InP/ZnS QDs induced ferroptosis and mitophagy by downregulating *gpx4b*, thereby inhibiting glutathione metabolism, consequently leading to retinal degeneration.

Did the downregulation of *prpf8* trigger glutathione metabolism dysfunction, or the inhibition of glutathione metabolism lead to the downregulation of *prpf8*? We found that *prpf8* mutation increased the expression level of unspliced *gpx4b* and decreased spliced *gpx4b* (Figure S11l,m, Supporting Information). Neither *gpx4b* mutation nor GSH supplement altered *prpf8* expression levels. These results suggested that *prpf8* regulated the splicing of *gpx4b* (Figure S12b,h; Figure S13f,g, Supporting Information).

Due to their extensive applications in LEDs, photovoltaics, displays, and biomedicine, these four types of QDs were selected for this study to investigate their potential visual developmental toxicity. Their pervasive use in manufacturing processes, products, and waste streams raises significant concerns regarding their biosafety and potential impact on environmental health. We observed that metallic QDs, such as InP/ZnS QDs and Mn:ZnS QDs, had a pronounced impact on ocular development, resulting in smaller eyes, thinner retinas, and abnormal visual responses. Whereas, BP QDs, one kind of non‐metal QDs, have no effect on zebrafish development. A previous study also showed that the non‐metal QDs have higher biosafety than metal‐containing QDs.^[^ ^64^ ^]^ These results hint that the development of non‐metal QDs is beneficial to human health and eco‐environmental protection.

Retinal degeneration is characterized by programmed cell death, progressive retinal damage, and thinner retinal layers.^[^ ^53^ ^]^ Retinal degenerative damage can be caused by vessel occlusion and light over‐simulation.^[^ ^35^ ^]^ Here, we examined the thickness of retinal layers of zebrafish at 72 hpf, 168 hpf, and even 21 dpf and found that InP/ZnS QDs exposure could decrease the retinal layers and cause progressive retinal damage. In agreement with our findings, titanium dioxide nanoparticle exposure also decreased the thickness of INL in mouse retina.^[^ ^3^ ^]^ Higher concentrations of airborne ultrafine particles were associated with the thinner retinal thickness from UK Biobank data.^[^ ^2^ ^]^ Besides, retinal degenerative diseases are usually along with vessel hyperplasia in retinas.^[^ ^45^, ^46^ ^]^ A recent epidemiologic study showed that urinary methyl paraben, an endocrine disrupter, was associated with wider retinal venular vessels in 4–6‐year‐old children from the ENVIRONAGE birth cohort.^[^ ^65^ ^]^ In our study, using two vascular endothelial cell Tg zebrafish, we observed that the retinal plexus vessels were significantly higher in InP/ZnS QDs exposure group compared with control. All these findings suggested that early‐life exposure to InP/ZnS QDs could cause retinal degenerative damage in zebrafish.

RPE plays multiple crucial roles in the retina, such as reception of photons, supporting the PR cells, maintenance of the barrier between blood and retina, transportation of nutrients and biomolecules, and phagocytosis of the damaged PR OS disc fragments caused by lights or some environmental pollutants.^[^ ^53^ ^]^ For example, SiO_2_ nanoparticles exposure at 150 µg mL^−1^ induced oxidative stress and apoptosis in human RPE cell line (ARPE‐19).^[^ ^66^ ^]^ Exposure to ZnS nanoparticles could cause apoptosis of primary mouse RPE cells.^[^ ^67^ ^]^ Here, we found that InP/ZnS QDs exposure decreased the number of RPE. Moreover, the damaged PR OS discs could not be wrapped. Due to the ultrafine size, nanoparticles can be internalized by RPE via endocytosis.^[^ ^68^ ^]^ In this study, InP/ZnS QDs were observed to be endocytosed by RPE using ICP‐MS and MSI. In line with our finding, a previous study presented that Ag_2_S QDs could be taken up by hepatic cell lines via endocytosis.^[^ ^69^ ^]^ We deduced that InP/ZnS QDs could be easily endocytosed by RPE cells and cause them death in zebrafish. Moreover, the damage of RPE resulted in lengthening the PR OS, which then possibly promoted the differentiation of PR(UV) to PR(RGB) in zebrafish.

In eukaryotic cells, the genes are usually interrupted by introns. The introns should be cut from messenger RNA precursors (pre‐mRNA), and the exons are spliced together to produce mature mRNA by spliceosome.^[^ ^50^ ^]^ There are many reports on the mutations in pre‐mRNA processing factors (such as PRPF3, 4, and 31) associated with retinal degeneration.^[^ ^70^, ^71^, ^72^ ^]^ For example, Li et al. reported that *prpf31* knockout shortened the eye size and caused the retinal degradation of zebrafish.^[^ ^72^ ^]^ In this study, we found that InP/ZnS QDs exposure also caused the decrease of *prpf8* expression in RPE of zebrafish. Mutation of *prpf8* showed the similar retinal toxicity as InP/ZnS QDs exposure. Moreover, *prpf8* overexpression could attenuate the retinal degenerative damage caused by InP/ZnS QDs. In agreement with our study, PRPF8 mutants in human also result in numerous splicing errors in RPE cells from retinal degeneration patients.^[^ ^73^ ^]^ But previous studies did not explain how *prpf8* induce retinal degeneration. In our study, through scRNA‐seq and subsequent verification, we found that the decrease of *prpf8* inhibited the splicing of *fth1a*, then induced ferroptosis. In addition, the downregulation of *prpf8* inhibited *gpx4b* splicing, which led to reduced glutathione metabolism, and caused ferroptosis and mitophagy of RPE (Figure 8i). Thereafter, InP/ZnS QDs exposure reduced the number and impaired the function of RPE, resulting in the elongation of PR OS and the increase of middle‐ and long‐wave cones [PR(RGB)]. Overall, these results suggest that the InP/ZnS QDs‐caused retinal degenerative damage could be co‐regulated by the RPE dysfunction and PR abnormal differentiation in zebrafish.

## 3 Conclusion

QDs have shown a great potential in many fields, including ophthalmology.^[^ ^10^ ^]^ However, there are limited reports on the effects of QDs to ocular tissues and cells. Through many approaches including scRNA‐seq, confocal imaging, OCT, TEM, MSI, gene mutation, and gene overexpression, our study shows that upon endocytosis by RPE, InP/ZnS QDs disrupt the *prpf8‐*related spliceosome and interfere with mRNA splicing, and then possibly lead to the accumulation of unspliced mRNA, including but not limited to *gpx4b*. The decreased expression of spliced *gpx4b* reduces the glutathione metabolism, which then causes mitophagy and aggravates ferroptosis of RPE. Moreover, overexpression of *prpf8* could attenuate the retinal degeneration caused by InP/ZnS QDs. Overexpression of *gpx4b* or supplement of GSH could totally rescue the retinal degeneration induced by InP/ZnS QDs. The spliceosome disruption may ultimately induce ferroptosis and mitophagy in RPE cells, subsequently resulting in the elongation of PR OS and promoting the differentiation of short‐wave cones [PR(UV)] to middle‐ and long‐wave cones [PR(RGB)]. Our work demonstrates that the typical metallic QDs may have potential health risk on ocular development, which makes an alarm for more caution to QD applications in industry and biomedicine. Our study provides valuable knowledge on the effects and mechanisms of metallic QDs on eye development, which would guide safer use of QDs in the future.

## 4 Experimental Section

### Shape and Size of QDs

The InP/ZnS PEG‐COOH QDs (WA‐26152‐10P), CdSe/ZnS PEG‐COOH QDs (WA21258), and Mn‐doped ZnS (Mn:ZnS) (WA‐41158‐2A) were purchased from Xingzi Nano (Shanghai, China). BP QDs were prepared according to our previous method.^[^ ^13^ ^]^ First, ten microliters of QDs solution were dropped onto a copper mash, and naturally dried overnight. Then, the shape and size were examined by TEM (Hitachi, Tokyo, Japan). The zeta potential and hydrodynamic size were determined by Malvern Zetasizer (Malvern Instruments Ltd, UK) instrument.

### Raman Shift

QDs (10 µL at 1 mg mL^−1^) were dropped on the silicon wafer, which was treated by absolute ethyl alcohol to Raman spectroscopy (Nanophoton, Japan). A solid‐state laser (Cobolt) whose wavelength was 437 nm was selected as the excitation source. To prevent damage, an ≈1 µm laser spot was focused which was held the laser power ≈0.1 mW on the sample. A grating of 1800 gr mm^−1^ and a charge coupled device cooled by nitrogen was equipped to a Horiba T64000 spectrometer. Under ambient conditions, the Raman spectra were recorded in backscattering geometry. Every sample acquired several sample spots for multiple spectra. For one sample, the spectra and degradation were not happened between different sample spots. The neon lines were used to calibrated the spectra.

### Zebrafish Culture and Exposure Methods

Guided by the Animal Ethics Committee of Xiamen University and routine procedures, the wild type TU zebrafish, Tg (*Atoh7:gapRFP::Ptf1a: GFP::Crx:CFPcaax*) zebrafish, Tg (*fli1:EGFP*) and Tg (*is5’:mCherry*) zebrafish were cultured under 14 h 10 h light/dark cycle. Temperature was 28 ± 1 °C, the pH was 7.0–7.4, and the dissolved oxygen was 7–8 mg L^−1^ in the zebrafish culture medium. Amounts of 0, 0.001, 0.01, 0.1, and 1 mg L^−1^ QDs were added to 5 mL zebrafish culture medium in six‐well plates, respectively. The culture medium contained 30 embryos per well. The exposure media were renewed every 12 h. The survival rate = the number of survivals / 30 × 100%. The hatching rate = the number of hatched larvae / the number of survivals × 100%. The malformation rate = the number of malformed larvae / the number of survivals × 100%. The eyes were imaged and measured using Leica M165 FC stereoscope (Frankfurt, Germany).

### Histology

Zebrafish larvae at 72 and 168 hpf were fixed by 4% paraformaldehyde (PFA). Before embedding in paraffin, the samples were dehydrated using by gradient of alcohol (75% overnight, 80% for 1 h, 95% for 50 min, 100% for 10 min twice, mixture of xylene and alcohol (1:1) for 20 min, xylene for 9 min and xylene for 5 min). The larvae were sliced into 5 µm sections, which were stained with hematoxylin for 10 s and eosin for 45 s. The photos were captured by a microscope (DM4B, Leica, Germany). The thicknesses of retinal layers were measured by ImageJ (Bio‐Rad, USA).

### OCT Imaging

The zebrafish was anesthetized by 0.016% 3‐aminobenzoic acid ethyl ester methanesulfonate (MS‐222, 98%, Sigma‐Aldrich, USA) for 5 min, then captured by OCT (Optoprobe OPIMG) to view the retinas.

### Bulk RNA‐Seq

For RNA‐seq experiments, retinas were collected from 20 zebrafish larvae with TRIzol reagent (Takara, China) with three biological replicates in each group. Illumina Sequencing (Novogene, China) was used to perform the RNA‐seq. STAR default was used to map the raw reads to the zebrafish genome (Zv10). The DEGs between two groups were identified by log2 (fold change) < −0.5 or > 0.5 and *p* value ≤ 0.05. The ClusterProfiler R package was used to perform the KEGG analysis.

### Real‐Time Quantitative PCR

The 20 zebrafish eyes were isolated and collected in 200 µL TRIzol reagent (Takara, Tokyo, Japan) at 72 hpf. Then, a SuperMix Kit (TransGen, Beijing, China) was used to synthesize the first‐strand cDNA. qPCR analysis was performed in an Mx3000P Real‐Time PCR system (Agilent Technologies, Santa Clara, USA). A reaction system and program based on our previous study were utilized.^[^ ^11^ ^]^ The 2^−△△Ct^ method was used to calculate the expression level. The reference gene was *β‐actin*. The primers used are listed in Table S2 (Supporting Information).

### Retina Preparation for scRNA‐Seq

According to a previous report,^[^ ^74^ ^]^ retinas were isolated from zebrafish at 72 hpf and put them in DMEM/F12 cell culture medium. Each group had 24 retinas from 12 larvae. Four milliliters of papain (Worthington), 8 µL L‐cysteine (12 mg mL^−1^, Sigma‐Aldrich) and 4 µL DNase (1%; Sigma‐Aldrich) were added into 184 µL DMEM/F12 (Invitrogen) to prepare the cell dissolved solution. The 100 mL washing buffer was prepared with 500 µL HEPES at 1 mol L^−1^ (Sigma‐Aldrich), 650 µL glucose (45%; Invitrogen), 5 mL fetal bovine serum (Gibco), and 93.85 mL PBS and stored at 4 °C. Then, the retinas were put into the cell dissolved solution at 37 °C for 15 min and gently up‐and‐down pipetted every 3 min. Further, 800 µL washing buffer was added to terminate the digestion. The single‐cell suspension was centrifuged at 500 g for 5 min after filtered using a 40 µL cell strainer (BD Falcon). Finally, the pellets were resuspended with 0.04% bovine serum albumin (BSA) dissolved by phosphate buffer solution (PBS). According to the manufacturer's protocol, ≈14 000 cells were loaded onto the Chromium Single Cell Chip (10 x Genomics). Trypan Blue staining was conducted to confirm the live cells over 93%.

### ScRNA‐Seq Data Processing

The single‐cell raw count matrices were generated by CellRanger files (Huada) to reference zebrafish genomes (Zv10). There were 27 373 and 28 540 reads per cell in control and InP/ZnS QDs group, respectively. The estimated number of cells were 14 319 and 13 909 in control and InP/ZnS QDs group, respectively. The raw count matrices were imported into R (4.1.1). Seurat 4.0 package (https://satijalab.org/seurat/) was used to analyze the single‐cell data matrices. The cells in the drop containing fewer than 200 genes (features) or the genes detected in less than three cells were filtered. Then, the Seurat object was created. The cells with the abnormal high expressing genes (features), the mitochondrial percentage over 10%, or the doublets cells were filtered. Then, a default size factor of 10 000 was used to Log normalize the filtered data. Finally, there were 7350 and 7133 cells in control and InP/ZnS QDs group, respectively, for following analysis. Principal component analysis (PCA) was used to perform the dimensionality reduction.

To cluster the cells, significant principal component (PCs) were used with the resolution at 0.5. Uniform Manifold Approximation and Projection (UMAP) was used to visualize the clustering results using the same PCs. Following, the cluster specific marker genes (the genes with significant differential expression in each cluster, logfc.threshold = 0.6, min.pct = 0.4) were identified by the FindAllMarkers in Seurat. The pseudotime analysis was performed by Monocle2 R package. The DEGs between two groups were identified by logfc.threshold = 0.25 and min.pct = 0.05. The KEGG pathway analysis was performed by “clusterProfiler” R package.

### RNA Velocyto Analysis

Annotation for the reads of spliced and unspliced mRNA, the velocyto.R command‐line tools were used to perform the read annotation. First, the annotation was initiated with BAM files from the CellRanger software. The CellRanger pre‐built packages were used to count the molecules, and separating them into “spliced,” “unspliced,” or “ambiguous.” The RNA velocity was calculated as previously reported.^[^ ^51^ ^]^ Finally, the calculated RNA velocity was mapped using Seurat.

### OKR Test

According to the previous report, performed the OKR test was performed.^[^ ^75^ ^]^ In order to explore the effects of InP/ZnS QDs on visual perception of zebrafish, zebrafish was exposed to 72 hpf with 0.1 and 1 mg L^−1^ QDs, and then continued to feed the larvae without InP/ZnS QDs to 168 hpf. The larvae were stimulated by Black‐White stripes, Blue‐White stripes, Green‐White stripes, and Red‐White stripes, respectively. At the same time, the eye positions of zebrafish were recorded by CCD camera. The videos were analyzed by ZebEyeTrack software.^[^ ^76^ ^]^ The ratio of eye velocity to stimulus velocity was used as a gain to evaluate the degree of OKR. Each test has six biological replicates.

### In Vitro Transcription and Microinjection

The gRNA for *prpf8* and *gpx4b* was synthesized according the manufacturer's protocol of TranscriptAid T7 High Yield Transcription Kit (Thermo Scientific, USA). Then, the gRNA and Cas9 protein were co‐injected to zebrafish embryos at one‐cell stage. As for the overexpression rescue experiments, *prpf8* and *gpx4b* were amplified open reading frame (ORF) from zebrafish cDNA, respectively, which were then cloned into pCS2‐CMV‐EGFP plasmids. The overexpression plasmids and empty plasmids were then injected into one‐cell stage zebrafish embryos at 50 ng µL^−1^, respectively. Then, embryos were exposed to 1 mg L^−1^ InP/ZnS QDs. At 72 hpf, the larvae were imaged and collected. Finally, the retinas were separated for qPCR. The sequences of *prpf8* gRNA and *gpx4b* gRNA are listed in Tables S3 and S5 (Supporting Information), respectively. The cloning primers for overexpression of *prpf8* and *gpx4b* are listed in Tables S4 and S6 (Supporting Information), respectively.

### Zebrafish Light‐Response Behavior Analysis

As previously reported, the zebrafish acquires swimming proficiency at 168 hpf.^[^ ^77^ ^]^ The 168‐hpf larvae were divided into 24‐well plates with one per well. For monochromatic light stimulation experiment, the swimming behavior was recorded in red, green, blue, and purple light, respectively, using the Noldus software (Wageningen, Netherlands).

### Imaging of Transgenic Zebrafish

The Tg (*Atoh7:gapRFP::Ptf1a:GFP::Crx:CFPcaax*) was kindly gifted from Dr. Jie He (Center for Excellence in Brain Science and Intelligence Technology, Chinese Academy of Sciences). The Tg (*is5’: mCherry*) zebrafish was purchased form the National Zebrafish Resource Center (Wuhan, China). The larvae at 72 or 168 hpf were anesthetized in low melting point agarose as above, and then imaged by a confocal microscope (LSM 900+Airyscans, Zeiss, Germany) or a light sheet microscope (Lightsheet 7, Zeiss, Germany).

### Immunofluorescence for PR Cells

The immunofluorescence was performed according a previous report.^[^ ^78^ ^]^ The 5 µm zebrafish retinal slices were incubated with anti‐OPN1MW1 (Abclonal, China), anti‐OPN1LW2 (Abclonal, China), anti‐OPN1SW1 (Abclonal, China), and anti‐RHO (Abcepta, China) solution (1:100), and anti‐acetylated‐α‐Tubulin (Abcam, ab24610), respectively, at 4 °C overnight. Then, the slices were washed by PBS for 5 min for three times. After that, the slices were incubated with the corresponding secondary antibody Alexa Fluor 647 (1:400, Affinity, China) for 1 h at 37 °C, and then washed with PBS for three times. As for TSA (Tyramide signal amplification) staining (cat: RK05880, ABclonal), different TSA stains were applied for 15 min after the incubation of the secondary antibody. Then, the nucleus was stained with 4',6‐diamidino‐2‐phenylindole (DAPI) for 3 min. The images were taken by a confocal microscope (LSM 900+Airyscans, Zeiss, Germany).

### Mass Spectrometry Imaging (MSI)

The paraffin sections were cut into 7‐µm slices, dried at 60 °C for 1 h, and rehydrated. Then, the metals [In(115) and (Ir(191)] were stained and imaged them using Hyperion mass spectrometer (Fluidigm, Canada). Ir(191) stained DNA to display the cell nuclei.

### Cell Viability

The (DMEM)/F12 medium (Gibco, NY, USA) with 10% fetal bovine serum (FBS) was used to culture the human retinal pigment epithelial (hRPE) cell line in the cell incubator with 5% CO_2_ at 37 °C. The cells were seeded and cultured in 96‐well plate at 1 × 10^4^ cells per well overnight. The cells were exposed to 0, 5, 10, 20, 40 mg L^−1^ InP/ZnS QDs for 24 h, respectively. Lately, MTT assay was conducted to calculate the cell viability (n = 6). By a microplate reader (Tecan Spark, Switzerland), absorbance values were measured at 490 nm.

### GSH Rescue Experiment at hRPE Cells

The hRPE cells were seeded and cultured in six‐well plate at ≈1 × 10^6^ cells per well overnight. hRPE cells were exposed by 20 mg L^−1^ InP/ZnS QDs with or without 5 mm GSH for 24 h.

### TEM for Zebrafish Retina and hRPE Cells

As previously reported,^[^ ^79^ ^]^ the 72 or 168‐hpf zebrafish larvae and the hPRE cells were fixed at 4 °C overnight. Then, the larvae were washed with PBS for three times. The samples were cut and imaged by FEI Tecnai TEM (Hillsboro, OR, USA).

### Mitochondrial Membrane Potential Detection

The hRPE cell mitochondrial membrane potential was detected by JC‐1 (Beyotime, China). According to the manufacturer's protocol, the hRPE cells were stained at 37 °C for 20 min. The inverted fluorescence microscope (Nikon, Japan) was used to capture the images.

### Lipid Peroxidation Measurement

The fluorescent probe C11 BODIPY 581/591 (Glpbio, USA) was used to stain the cell lipid peroxidation. The cells were incubated with the probe at 37 °C for 30 min. After that, the green marked the oxidized cell membrane, and the red marked normal. The inverted fluorescence microscope (Nikon, Japan) was used to capture the images.

### Western Blotting

The sodium dodecyl sulfate (SDS) loading buffer was used to lyse the hRPE cells in the six‐well plate after treatment. Then SDS‐polyacrylamide gel electrophoresis gel was used to load the lysates. After that, polyvinylidene fluoride membranes were used to transfer the proteins. The protein was blocked by skim milk. After that, the primary antibodies, including anti‐GAPDH (ABclonal, AC033), anti‐LC3A/B (Cell Signaling Technology, 4108S), anti‐PINK1 (ABclonal, A24745), anti‐GPX4 (Beyotime, AF7020), and anti‐PRPF8 (ABclonal, A4575) were used to incubate the proteins. Further, the primary antibody was bound to IgG‐AP‐linked secondary antibody (Millipore, USA). Finally, the enhanced chemiluminescent substrate (Pierce, Rockford, USA) was used to develop the blot, which was captured by chemiluminescence imaging equipment (Bio‐Rad, USA). The ImageJ software (Bio‐Rad, USA) was used to quantify the band intensity.

### Data Analysis

Mean ± standard error (SE) was used to presented the data. One‐way ANOVA followed by *Duncan test* was used to assess the statistical significance. The different letters were used to indicate the significantly different which the means of *p* < 0.05.

## Conflict of Interest

The authors declare no conflict of interest.

## Supporting information



Supporting Information

## Data Availability

The data that support the findings of this study are available from the corresponding author upon reasonable request.
