# Supplementary material for: Quantum Dots‐caused Retinal Degeneration in Zebrafish Regulated by Ferroptosis and Mitophagy in Retinal Pigment Epithelial Cells through Inhibiting Spliceosome
Source: Adv Sci (Weinh). 2024 Oct 17;11(46):2406343. doi: 10.1002/advs.202406343 (PMC11633537; doi:10.1002/advs.202406343)
Supplement: Supplementary file 1 — Supporting Information [file ADVS-11-2406343-s001.pdf]

ADVANCED SCIENCE
Open Access

Supporting Information

for *Adv. Sci.*, DOI 10.1002/advs.202406343

Quantum Dots-caused Retinal Degeneration in Zebrafish Regulated by Ferroptosis and Mitophagy in Retinal Pigment Epithelial Cells through Inhibiting Spliceosome

Naying Zheng, Tingting Liao, Chuchu Zhang, Zheyang Zhang, Sen Yan, Xiaohan Xi, Fengkai Ruan, Chunyan Yang, Qingliang Zhao, Wenbo Deng, Jialiang Huang, Zi-Tao Huang, Zhi-Feng Chen, Xiang Wang, Qingming Qu, Zhenghong Zuo and Chengyong He**

Supplementary files

Quantum Dots-Caused Retinal Degeneration in Zebrafish Regulated by Ferroptosis and Mitophagy in Retinal Pigment Epithelial Cells through Inhibiting Spliceosome

Naying Zheng[1], Tingting Liao[1], Chuchu Zhang[1], Zheyang Zhang[1], Sen Yan[2], Xiaohan Xi[2], Fengkai Ruan[1], Chunyan Yang[1], Qingliang Zhao[3], Wenbo Deng[4], Jialiang Huang[1], Zi-Tao Huang[5], Zhi-Feng Chen[5], Xiang Wang[2], Qingming Qu[1], Zhenghong Zuo[1], Chengyong He[1]**

[1] Department of Ophthalmology in Xiang'an Hospital of Xiamen University, State Key Laboratory of Cellular Stress Biology, School of Life Sciences, Faculty of Medicine and Life Sciences, Xiamen University, Xiamen, Fujian 361102, China

[2] Department of Chemistry, State Key Laboratory of Physical Chemistry of Solid Surfaces, Collaborative Innovation Center of Chemistry for Energy Materials (i-ChEM), Innovation Laboratory for Sciences and Technologies of Energy Materials of Fujian Province (IKKEM), College of Chemistry and Chemical Engineering, Xiamen University, Xiamen 361005, China

[3] State Key Laboratory of Vaccines for Infectious Diseases, Center for Molecular Imaging and Translational Medicine, Xiang An Biomedicine Laboratory, School of Public Health, Xiamen University, Xiamen, Fujian, 361005, China.

[4] Key Laboratory of Reproductive Health Research, Fujian Province University, School of Medicine, Xiamen University, Xiamen, Fujian, 361005, China.

[5] Guangdong Key Laboratory of Environmental Catalysis and Health Risk Control, Guangdong-Hong Kong-Macao Joint Laboratory for Contaminants Exposure and Health, School of Environmental Science and Engineering, Guangdong University of

Technology, Guangzhou 510006, China

* Corresponding author, E-mail: zuozhenghong@xmu.edu.cn & hecy@xmu.edu.cn.

Table S1 Characterizations of QDs.

QDs	Size[a)] (nm)	Hydrodynamic size[b)] (nm)	Zeta potential[c)] (mV)
CdSe/ZnS QDs	16.73 ± 3.1	90.61 ± 32.39	-17.93 ± 0.85
Mn:ZnS QDs	2.21 ± 2.5	618.27 ± 17.62	-14.57 ± 1.19
InP/ZnS QDs	6.21 ± 2.1	120.20 ± 45.51	-7.0 ± 1.38
BP QDs	5.61 ± 2.4	247 ± 23.58	-0.30 ± 0.84

a) The primary size of different QDs was measured by the TEM (Hitachi HT-7800, Japan); b-c) the hydrodynamic size b) and zeta potential c) were measured in zebrafish culture medium.

Table S2 The primers used for qPCR.

Gene symbol	Primer sequence (5'-3')	Genbank number	Product length (base pairs, bp)
β-actin	F: AAGCAGGAGTACGATGAGTC	NM_181601.5	238
	R: TGGAGTCCTCAGATGCATTG		
slc7a11	F: TCTGGCTTTCTACTCGGGGA	XM_009291228.3	212
	R: AGGTCACAGCAACAGCGTTA		
gnat2	F: CACCTCCACGTAGGTCACAC	NM_131869.2	183
	R: GCTACCCATCTCGTCGTCTG		
grk7a	F: AGGCATCTCCGTTCTTCGAC	NM_001031841.3	295
	R: CAGAGGTGGGTCTTGGTGTC		
rpl35	F: TGACCGCTGCAACTTCCTTT	NM_173233.2	200
	R: ACTTGCGGACAACACGGATT		
rps23	F: AGAAAACTGCGGAACCACCG	NM_001110121.1	296
	R: CCTTACGACCGAATCCTGCC		
srsf5a	F: GTCGTGTGTTTATTGGCCGT	NM_200867.1	285
	R: GGGACTGACGATAACCTCCA		
prpf8	F: CACTGGCGAGAAACTGCTTT	NM_200976.2	295

	R: TCCTGTTCGTCATGTCACCA		
gpx4b u	F: TAGCATGACCATTGACAGTTTCC	NC_007113.7	200
	R: CCAGAGAGAACAGAGCCCAA		
gpx4b s	F: CAATGTGTGCCCAAGCCAAT	NM_001030070.2	222
	R: GGAAGCCCAGGATGCGTAAA		
fth1a u	F: TGAGCCACTCCTTTTAGCCT	NM_131585.1	293
	R: CGTGAGCTTGTCGAACATGT		
fth1a s	F: AACCAGAGAAGGACGAGTGG	NC_007118.7	263
	R: CGTGAGCTTGTCGAACATGT		
opn1sw1	F: CTTGCGGACCAGATTACTACACACT	NM_131253.2	195
	R: TCACTTCCCTCTCAGCCTTCT		
opn1mw1	F: GTGTAATGGAGGGATTCTTCG	NM_131253.2	250
	R: GTGTAGTAATCTGGTCCGCAAG		
opn1lw2	F: CTGCTGCAAACCCAGGCTAT	NM_001002443.2	259
	R: TCCAGTTCTTCCCTCTTGTTCA		
PRPF8	F: GTGAGGGCCTGTGGGATTCT	NM_006445.4	257
	R: GGTCATGTCTCCATGGTCTCG		
GPX4	F: GAGGCAAGACCGAAGTAAACTAC	NM_001367832.1	100
	R: CCGAACTGGTTACACGGGAA		

Table S3 The sequences of gRNA for *prpf8*.

**gRNA number**	**Primer sequence (5'-3')**
gRNA-1	TAATACGACTCACTATAGGGAGGAACACCTGGAGGCATGTTTTAGAGCTAGAAATAGC
gRNA-2	TAATACGACTCACTATAGGTCCAGGAAGACCTCTGTAGGTTTTAGAGCTAGAAATAGC
gRNA-3	TAATACGACTCACTATAGGGAAGACCTCTGTAGGGGAAGTTTTAGAGCTAGAAATAGC
gRNA-4	TAATACGACTCACTATAGGCAGCTCCTTTCCCCTACAGGTTTTAGAGCTAGAAATAGC
gRNA-5	TAATACGACTCACTATAGGGTCCAGGAAGACCTCTGTAGTTTTAGAGCTAGAAATAGC
gRNA-6	TAATACGACTCACTATAGGGAGGAACACCTGGAGGCATGTTTTAGAGCTAGAAATAGC

Table S4 The primers for *prpf8* cloning.

Usage	Primer sequence (5'-3')
ORF cloning	F: ATGGCAGCTCCTTTCCCCTA
	R: TGCATACATATCCTCACGGTCAG
Clone to vector	F: GTCGGAGCAAGCTTGCCACCATGGCAGCTCCTTTCCCCTA
	R: CCCTTGCTCACCATGGATCCTGCATACATATCCTCACGGTCAG

Table S5 The sequences of gRNA for *gpx4b*.

gRNA number	Primer sequence (5'-3')
gRNA-1	TAATACGACTCACTATAGGGGAGCGCTGTTGGTCGGAGGTTTTAGAGCTAGAAATAGC
gRNA-2	TAATACGACTCACTATAGGGGCTGTTGGTCGGAGCGGTGTTTTAGAGCTAGAAATAGC
gRNA-3	TAATACGACTCACTATAGGGCGCTGTTGGTCGGAGCGGGTTTTAGAGCTAGAAATAGC
gRNA-4	TAATACGACTCACTATAGGGTGTTTCAGAGAGCGCTGTGTTTTAGAGCTAGAAATAGC

Table S6 The primers for *gpx4b* cloning.

Usage	Primer sequence (5'-3')
Clone to vector	F: GGGACGTCGGAGCAAGCTTGCCACCATGTGGTTGTTTCAGAGAGC
	R: CCTCGCCCTTGCTCACCATGGATCCCTACAGGTACTTGGGAAGAT

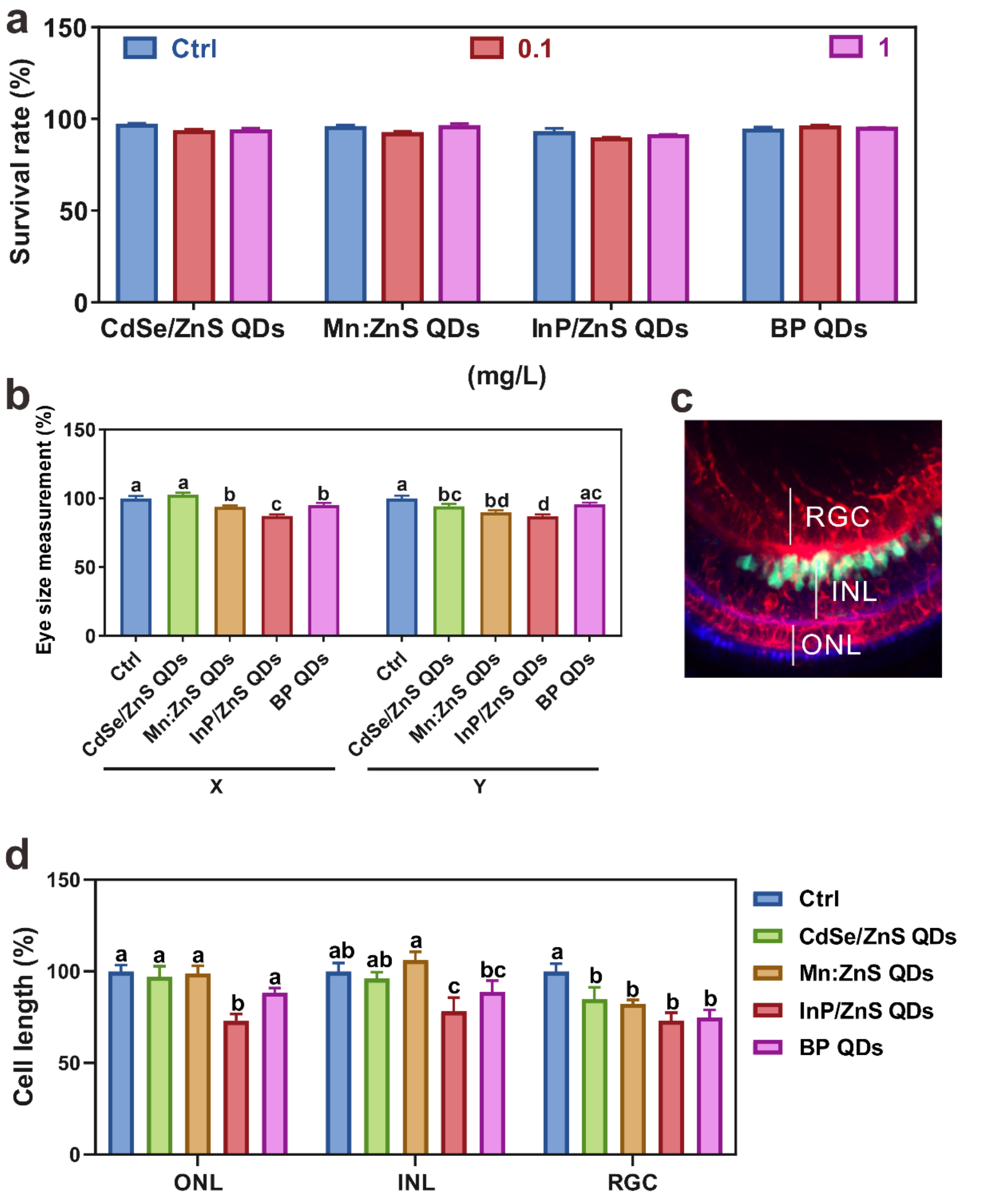

Figure S1 The developmental effects of QD exposure to zebrafish embryos from 0.5 to 72 hpf. a) The survival rate at 72 hpf after different QDs exposure, n ≥ 4. b) the eye size at 72 hpf after different QDs exposure, n ≥ 14. c) Schematic illustration of the different layer of retinal cells. d) The different layer of retinal length measured at 168 hpf after different QDs exposure, n ≥ 4. The data are presented as Mean ± SE. Significant change among different groups ($p < 0.05$) is indicated by the different letters on the bar.

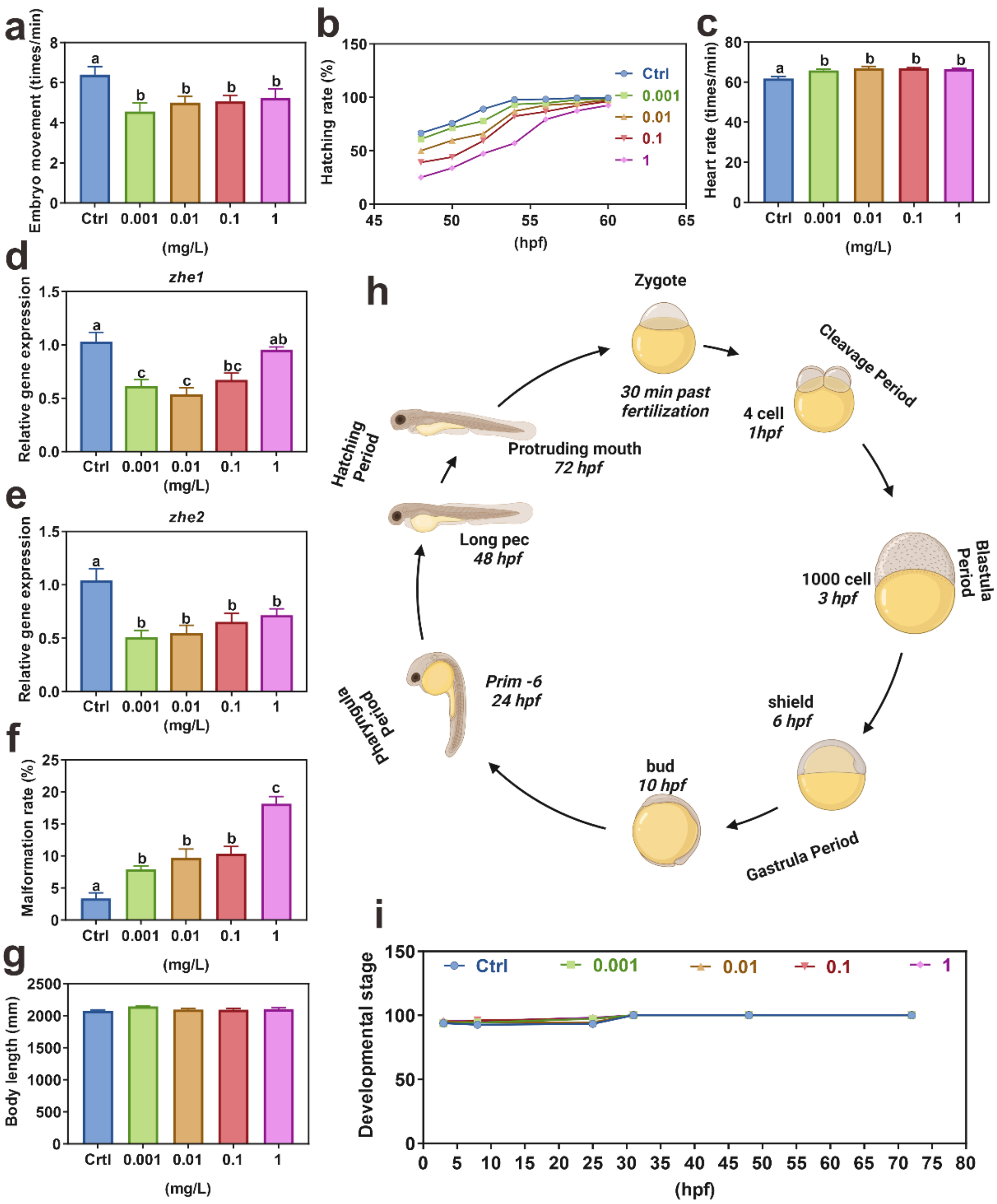

Figure S2. The developmental effects of InP/ZnS QDs in zebrafish. a) The embryo movements of zebrafish embryo at 24 hpf, n = 6. b) The hatching rate of zebrafish larvae from 48 to 72 hpf, n = 6. c) The heart rate of zebrafish larvae at 72 hpf, n = 6. d-e) The relative gene expression of *zhe1* and *zhe2*, n ≥ 5. f) The malformation rate of zebrafish larvae at 72 hpf, n = 6. g) The body length of zebrafish at 72 hpf, n ≥ 16. h) The schematic showed the developmental stage of zebrafish from 0.5 to 72 hpf. i) The developmental stage statistics from 0.5 to 72 hpf, n = 6. The data are presented as Mean ± SE. Significant change among different groups ($p < 0.05$) is indicated by the different letters on the bar.

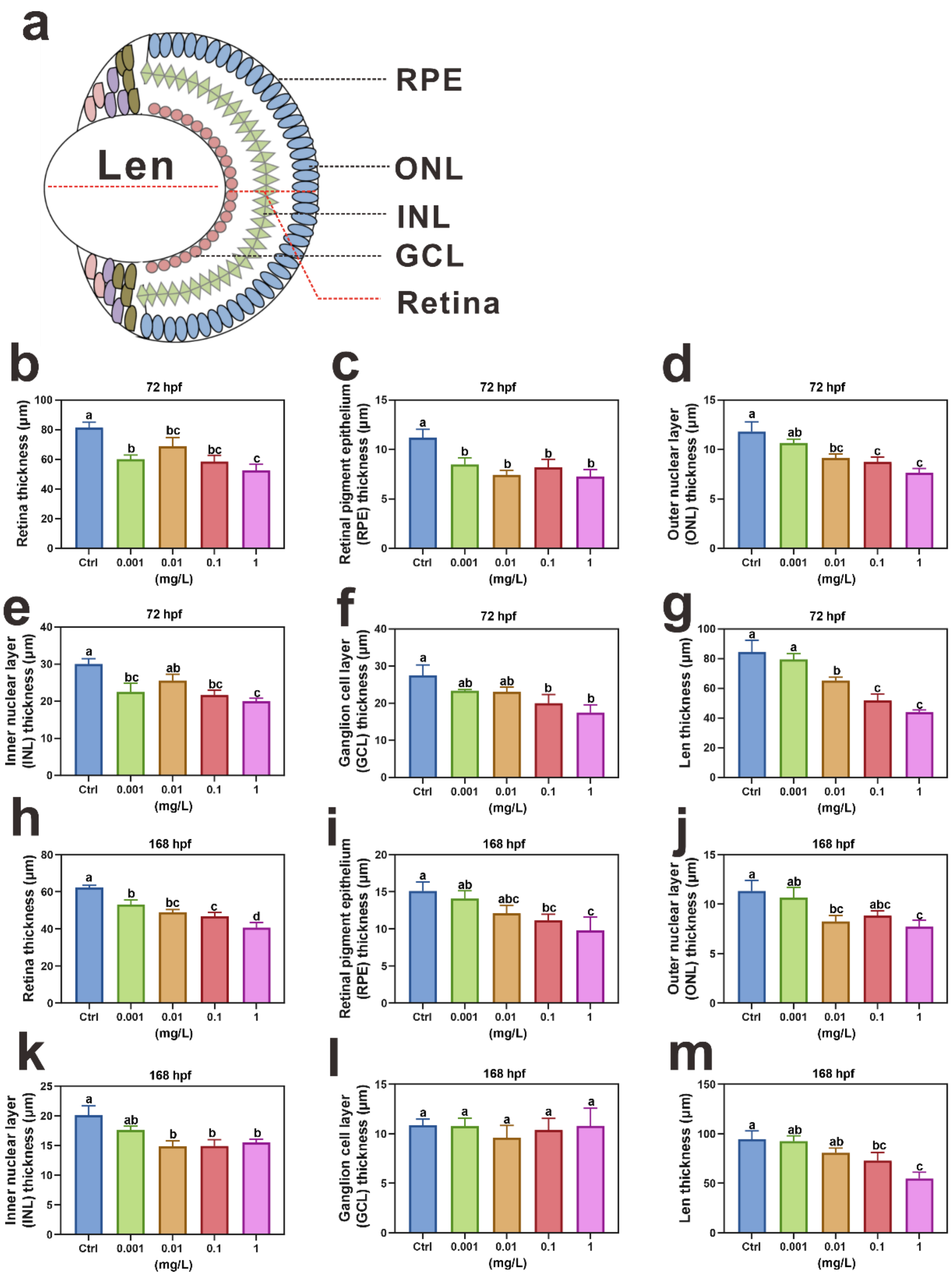

Figure S3. The thicknesses of different retinal layers of zebrafish exposure to InP/ZnS QDs at 72 hpf and 168 hpf. a) The schematic diagram of zebrafish eye. b-m) The thicknesses of retina b), different retinal layers, including the retinal pigment epithelium (RPE) c), the outer nuclear layer (ONL) d), the inner nuclear layer (INL) e), the retinal ganglion cell layer (GCL) f), and the len g) were measured at 72 hpf. h) The thicknesses of retina h), RPE i), ONL j), INL k), GCL l), and len m) were measured at

168 hpf, n ≥ 4. The data are presented as Mean ± SE. Significant change among different groups ($p < 0.05$) is indicated by the different letters on the bar.

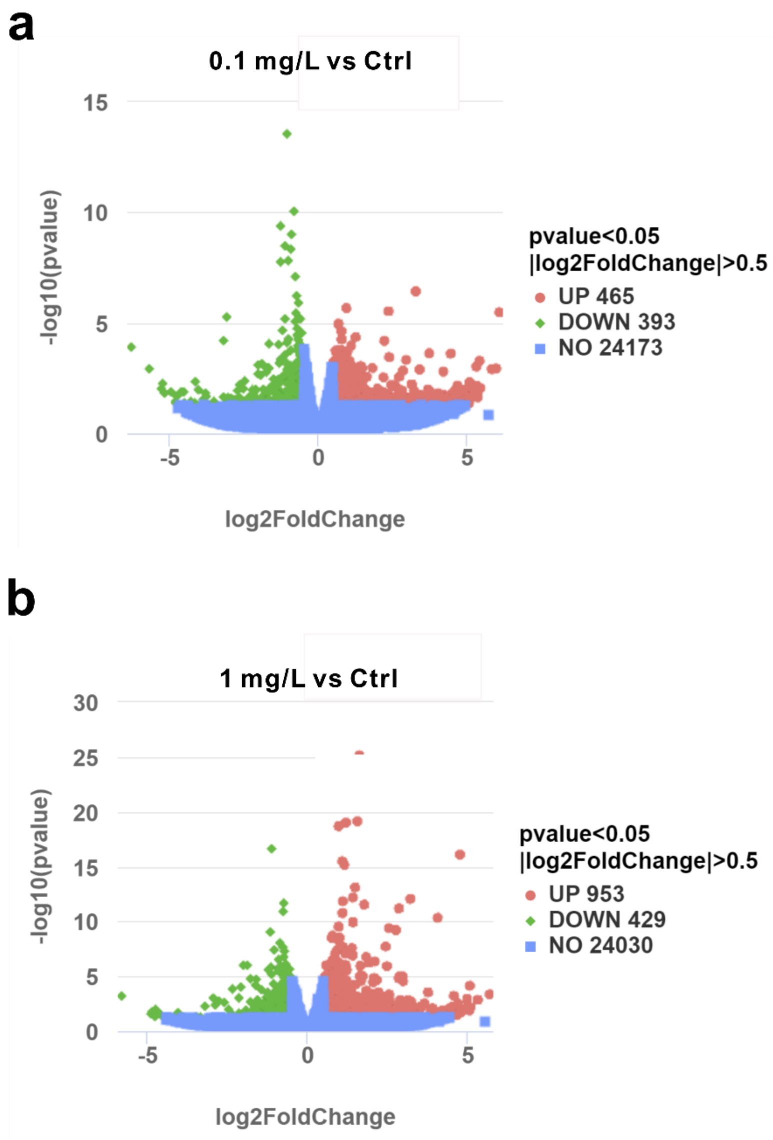

Figure S4. The volcano plot of the differential expressed genes (DEGs) in zebrafish larvae exposure to InP/ZnS QDs at 72 hpf. The volcano plot of the DEGs with 0.5-fold cutoff and a $p < 0.05$ in 0.1 a) and 1 mg L^{-1} b) groups, respectively.

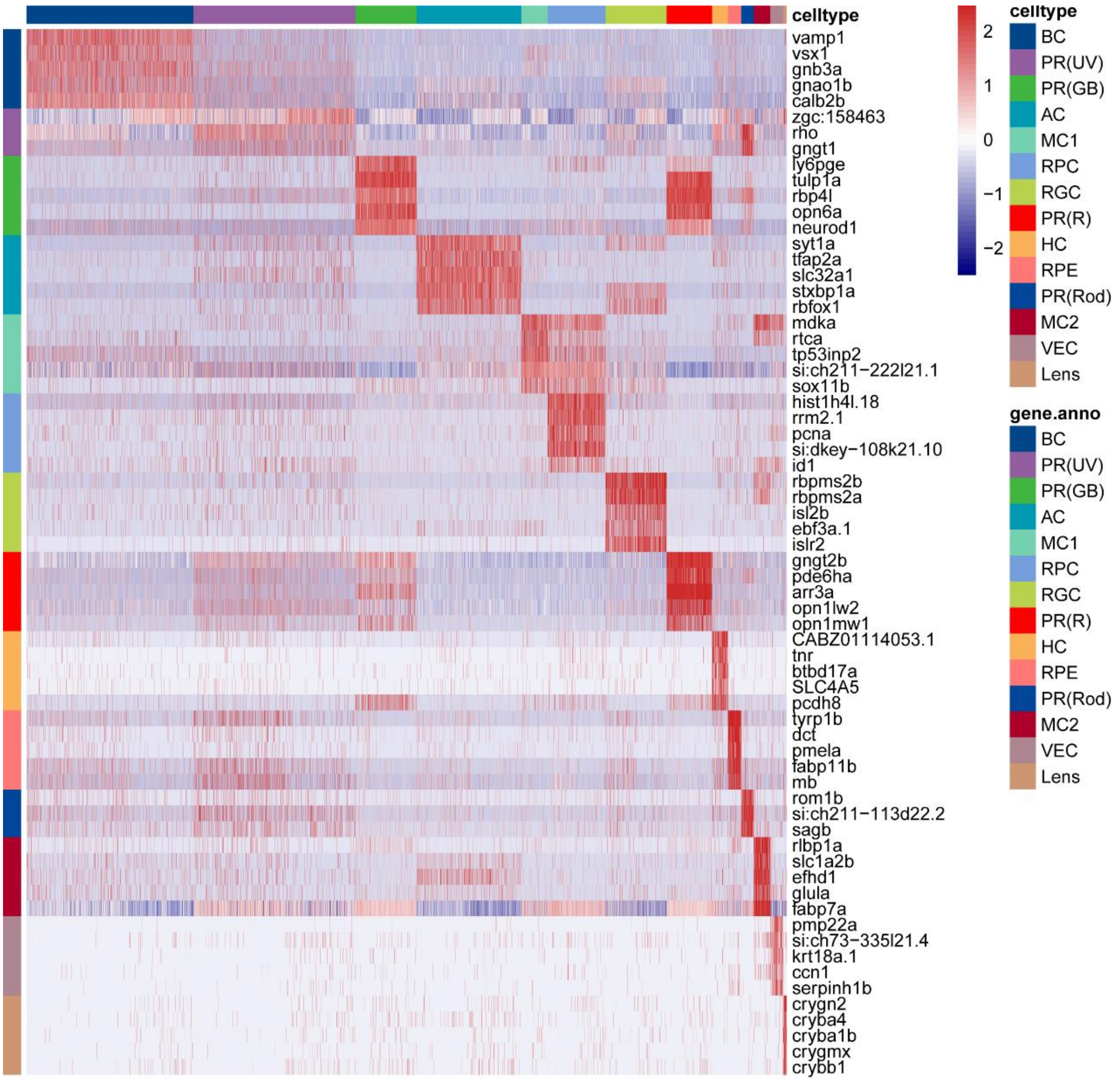

Figure S5. The heatmap of top five marker genes for each cluster in all retinal cells basing on the scRNA-seq for 1 mg L^{-1} InP/ZnS QDs exposure from 0.5 to 72 hpf.

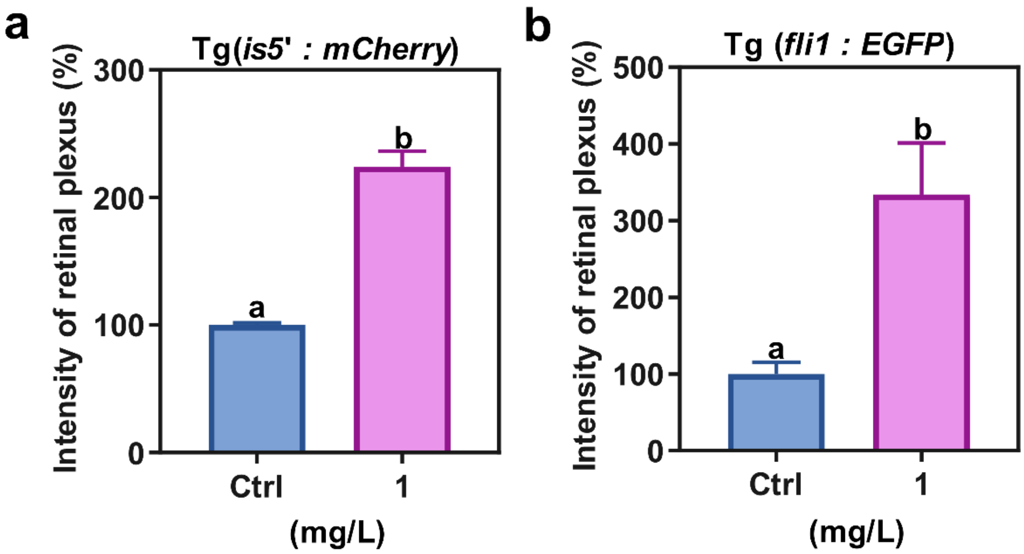

Figure S6. The fluorescence intensity of retinal plexus after exposure to InP/ZnS QDs at 72 hpf. a) The fluorescence intensity of Tg(*is5' : mCherry*) zebrafish retinal plexus. b) The fluorescence intensity of Tg(*fli1 : EGFP*) zebrafish retinal plexus, n = 3. The data are presented as Mean ± SE. Significant change among different groups ($p < 0.05$) is indicated by the different letters on the bar.

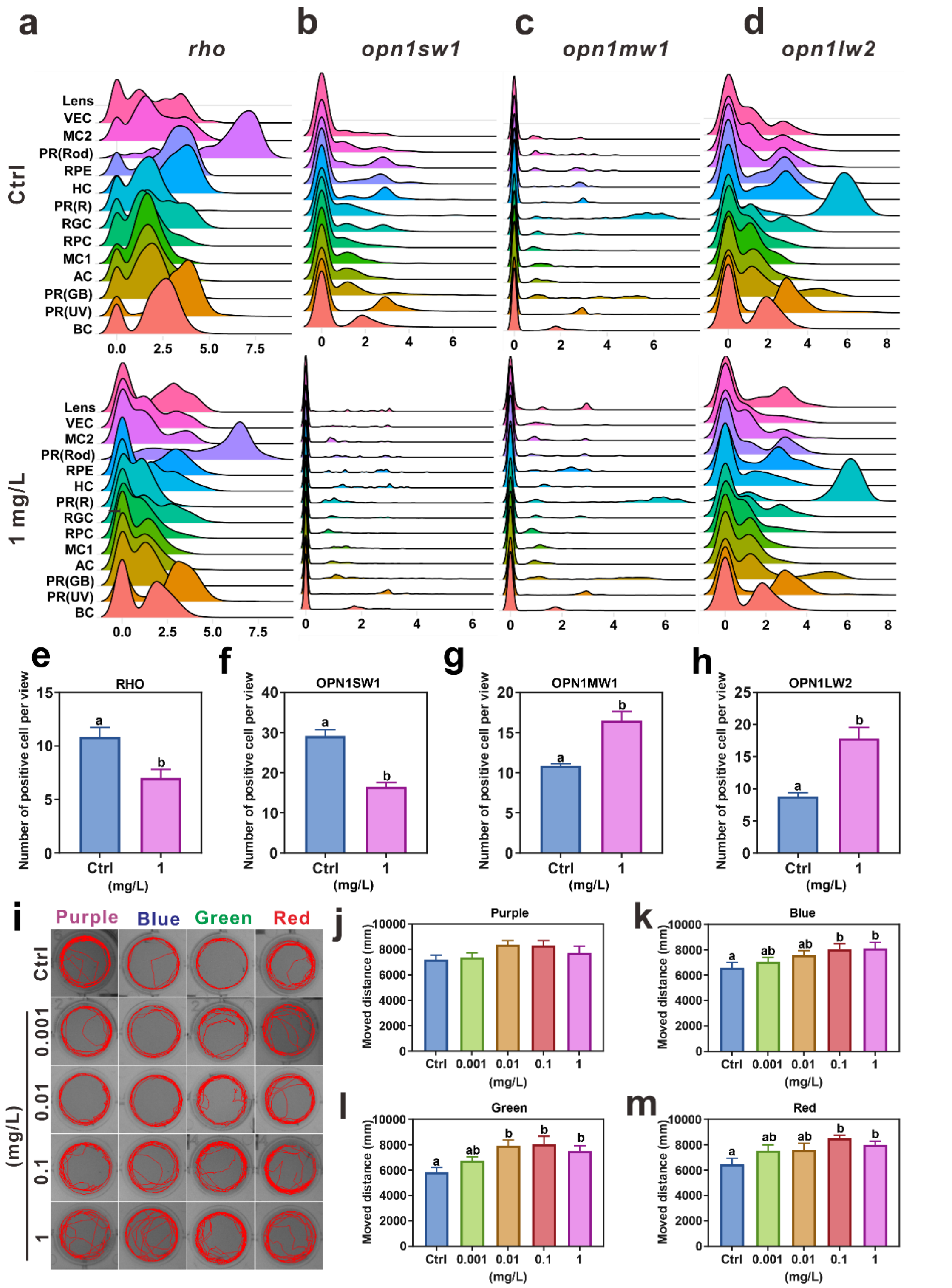

Figure S7. InP/ZnS QDs exposure induced zebrafish more sensitive to red, green, and blue lights. The relative expression level of genes of *rho* a), *opn1sw1* b), *opn1mw1* c) and *opn1lw2* d) in control and InP/ZnS QDs group. e-h) The number of RHO e), OPN1SW1 f), OPN1MW1 g), and OPN1LW2 h) positive cells per visual field at 72 hpf, n = 6. i) Visual responses of 168-hpf zebrafish exposed to different-wavelength

lights. The moved distance of zebrafish under purple j), blue k), green l), and red m) light stimulation, respectively, n ≥ 21. The data are presented as Mean ± SE. Significant change among different groups ($p < 0.05$) is indicated by the different letters on the bar.

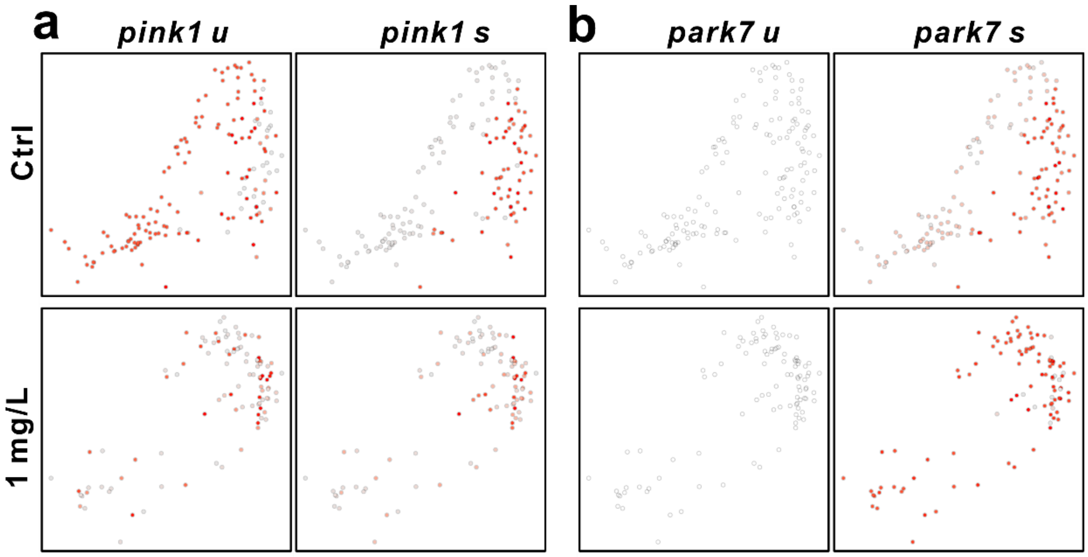

Figure S8. Effects of InP/ZnS QDs **exposure on splicing levels of mitophagy-related genes.** The unspliced and spliced levels of *pink1* a) and *park7* b) mRNAs.

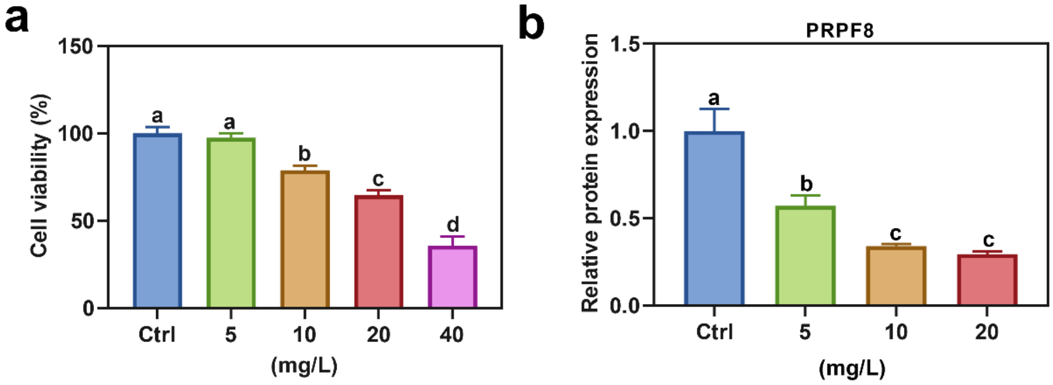

Figure S9. InP/ZnS QDs damaged hRPE cells in a concentration-dependent manner. a) The cell viability of hRPE cells after InP/ZnS QDs treatment, n = 6. b) The relative protein expression level of PRPF8 in hPRE cells after InP/ZnS QDs exposure, n = 3. The data are presented as Mean ± SE. Significant change among different groups ($p < 0.05$) is indicated by the different letters on the bar.

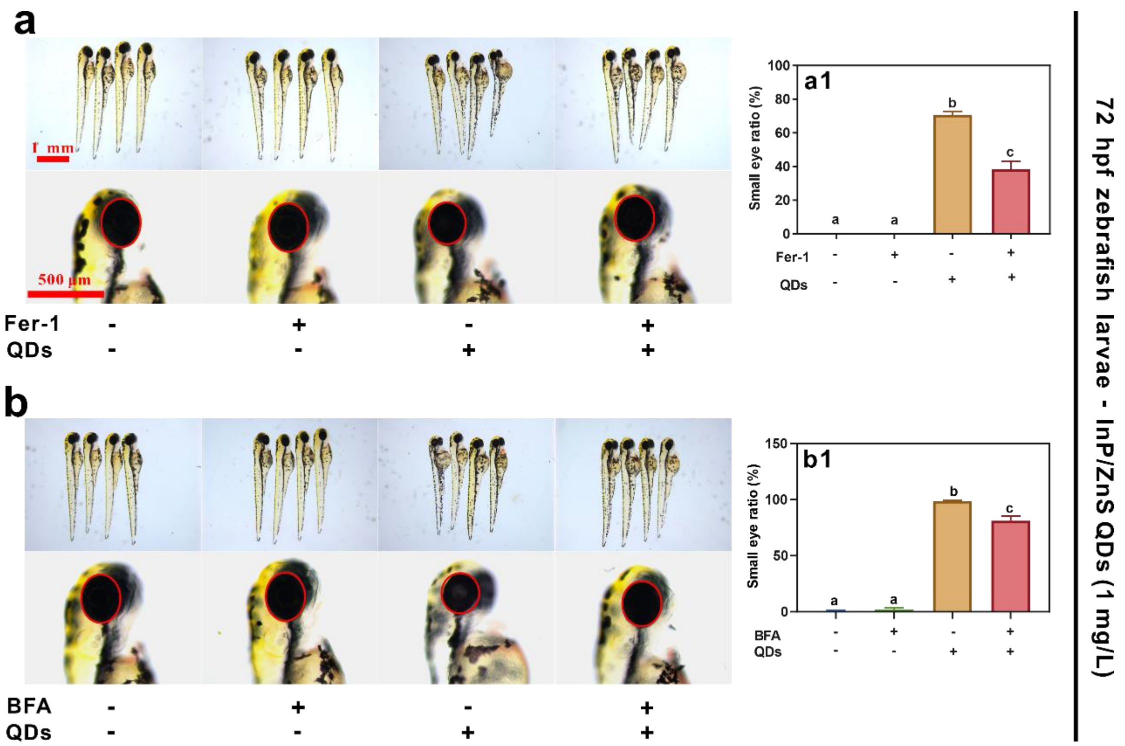

Figure S10. The roles of ferroptosis and mitophagy in zebrafish retinal degeneration caused by InP/ZnS QDs. a) Fer-1 (1 µM) partly rescued the small eye size caused by InP/ZnS QDs at 72 hpf. The wild type embryos were divided four groups: Control, Fer-1 control, InP/ZnS QDs and InP/ZnS QDs with Fer-1 groups. Scale bar is 1 mm and 500 µm, respectively. The small eye ratio a1), n = 6. b) BFA (2 µg/mL) partly rescued the small eye size caused by InP/ZnS QDs at 72 hpf. The wild type embryos were divided four groups: Control, BFA, InP/ZnS QDs and InP/ZnS QDs with BFA groups. Scale bar is 1 mm and 500 µm, respectively. The small eye ratio b1), n = 5-6. The data are presented as Mean ± SE. Significant change among different groups ($p < 0.05$) is indicated by the different letters on the bar.

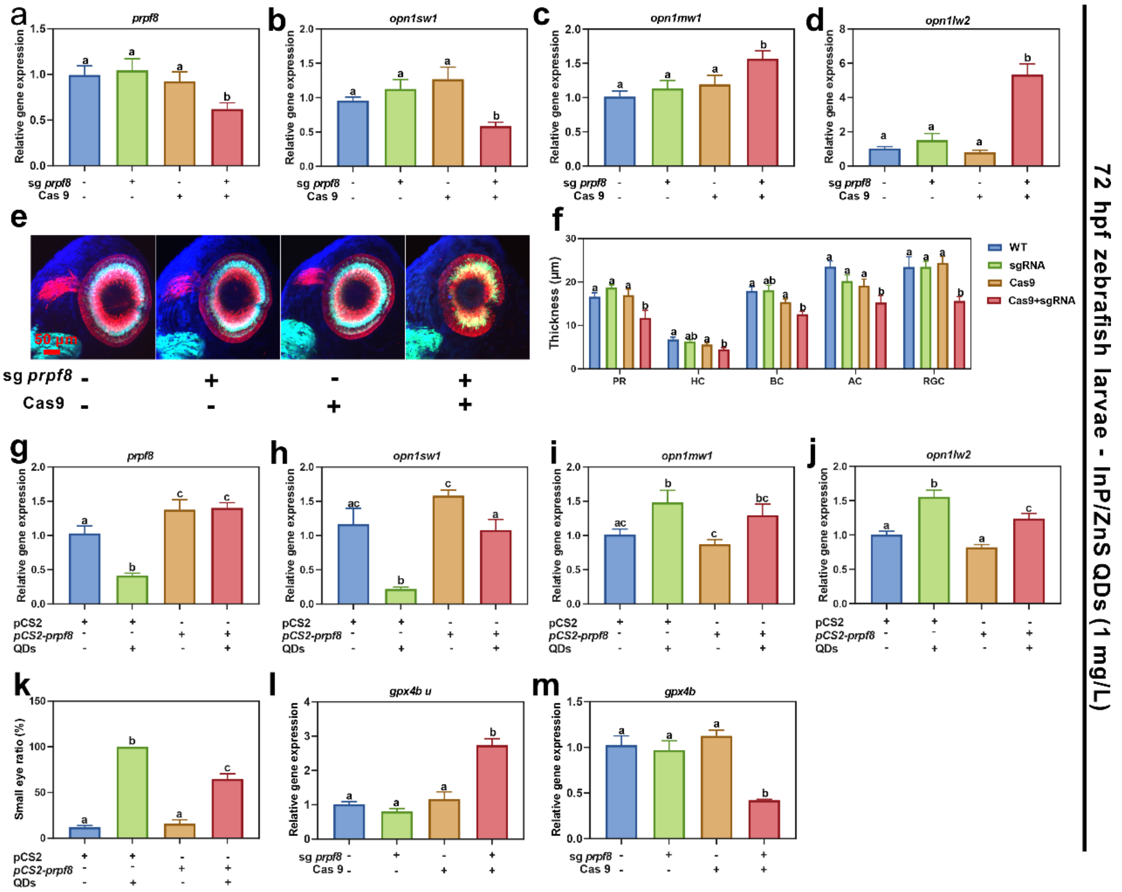

Figure S11. The role of *prpf8* in zebrafish retinal degeneration caused by InP/ZnS QDs. a-d) The relative expression level of *prpf8* a), *opn1sw1* b), *opn1mw1* c), and *opn1lw2* d) from the groups of no injection control, injection with Cas9 protein, injection with sgRNAs for *prpf8*, and co-injection of Cas9 and sgRNAs for *prpf8*, n = 4-6. e) The lateral views of the effects of *prpf8* mutation on retina in Tg (*Atoh7:gapRFP::Ptf1a:GFP::Crx:CFPcaax*) zebrafish at 72 hpf. The embryos were also divided four groups as wild type. Scale bar, 50 μm. f) The thickness of different retinal layers was measured at 72 hpf in Sofa Tg zebrafish, n = 4. The relative expression level of *prpf8* g), *opn1sw1* h), *opn1mw1* i), *opn1lw2* j), unspliced *gpx4b* l) and spliced *gpx4b* m) at 72 hpf after injection with InP/ZnS QDs exposure. The wild type embryos were divided four groups: injection of pCS2 plasmids with or without InP/ZnS QDs exposure and injection of pCS2-*prpf8* plasmids with or without InP/ZnS QDs exposure, n ⩾ 4. k) *prpf8* overexpression partially rescued the small eye ratio caused by InP/ZnS QDs at 72 hpf, n = 6. The data are presented as Mean ± SE.

Significant change among different groups ($p < 0.05$) is indicated by the different letters on the bar.

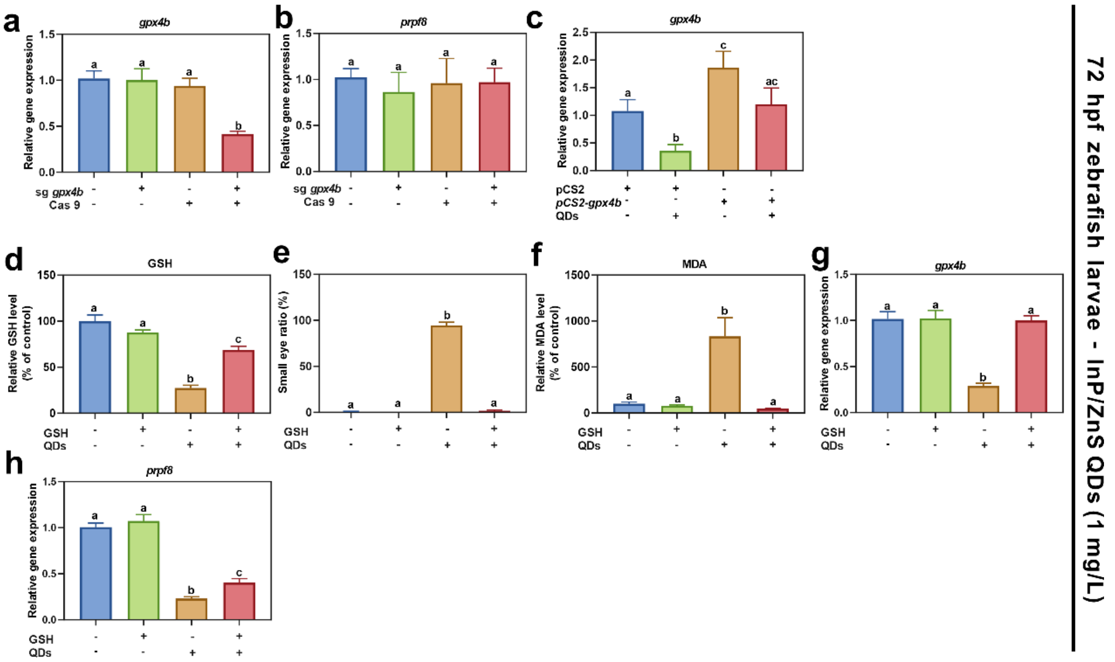

Figure S12. The role of glutathione metabolism pathway in zebrafish retinal degeneration caused by InP/ZnS QDs. a-b) The relative expression level of *gpx4b a), and prprf8 b)* from the groups of no injection control, injection with Cas9 protein, injection with sgRNA for *gpx4b*, and co-injection of Cas9 and sgRNA for *gpx4b*, n = 5-6. c) The relative expression level of *gpx4b* at 72 hpf after injection with exposure. The wild type embryos were divided four groups: injection of pCS2 plasmids with or without InP/ZnS QDs exposure and injection of pCS2-*gpx4b* plasmids with or without InP/ZnS QDs exposure, n = 5-6. d-h) The rescue effect of GSH to InP/ZnS QDs exposure. The relative GSH d) and MDA f) levels, n = 3. e) The small eye ratios, n = 6. The relative gene expression levels of *gpx4b* g) and *prpf8* h), n = 5-6. The data are presented as Mean ± SE. Significant change among different groups ($p < 0.05$) is indicated by the different letters on the bar.

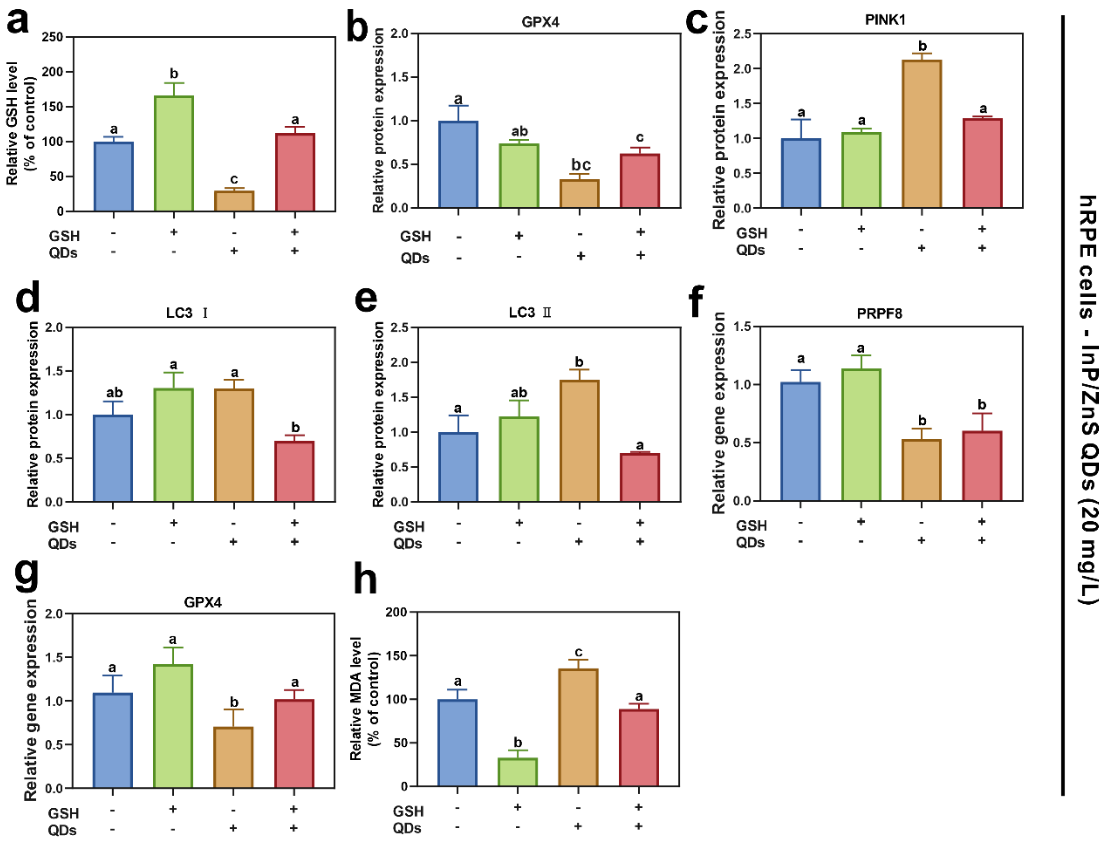

Figure S13. GSH rescued the mitophagy and ferroptosis in hRPE cells caused by InP/ZnS QDs. a) The relative GSH levels of hRPE cells after InP/ZnS QDs exposure with or without GSH, n = 3. The relative protein expression of GPX4 b), PINK1 c), LC3 I d) and LC3 II e), n = 3. The relative gene expression of PRPF8 f) and GPX4 g), n = 5-6. h) The relative MDA levels of hRPE cells after InP/ZnS QDs exposure with or without GSH, n = 3. The data are presented as Mean ± SE. Significant change among different groups ($p < 0.05$) is indicated by the different letters on the bar.
